# The social and structural architecture of the yeast protein interactome

**DOI:** 10.1038/s41586-023-06739-5

**Published:** 2023-11-15

**Authors:** André C. Michaelis, Andreas-David Brunner, Maximilian Zwiebel, Florian Meier, Maximilian T. Strauss, Isabell Bludau, Matthias Mann

**Affiliations:** 1 Max-Planck Institute of Biochemistry, Martinsried, Germany; 2 Drug Discovery Sciences, Boehringer Ingelheim Pharma, Biberach Riss, Germany; 3 Functional Proteomics, Jena University Hospital, Jena, Germany; 4 NNF Center for Protein Research, University of Copenhagen, Copenhagen, Denmark

**Keywords:** Protein-protein interaction networks, Mass spectrometry, Systems analysis, Molecular modelling

## Abstract

Cellular functions are mediated by protein–protein interactions, and mapping the interactome provides fundamental insights into biological systems. Affinity purification coupled to mass spectrometry is an ideal tool for such mapping, but it has been difficult to identify low copy number complexes, membrane complexes and complexes that are disrupted by protein tagging. As a result, our current knowledge of the interactome is far from complete, and assessing the reliability of reported interactions is challenging. Here we develop a sensitive high-throughput method using highly reproducible affinity enrichment coupled to mass spectrometry combined with a quantitative two-dimensional analysis strategy to comprehensively map the interactome of *Saccharomyces cerevisiae*. Thousand-fold reduced volumes in 96-well format enabled replicate analysis of the endogenous GFP-tagged library covering the entire expressed yeast proteome^1^. The 4,159 pull-downs generated a highly structured network of 3,927 proteins connected by 31,004 interactions, doubling the number of proteins and tripling the number of reliable interactions compared with existing interactome maps^2^. This includes very-low-abundance epigenetic complexes, organellar membrane complexes and non-taggable complexes inferred by abundance correlation. This nearly saturated interactome reveals that the vast majority of yeast proteins are highly connected, with an average of 16 interactors. Similar to social networks between humans, the average shortest distance between proteins is 4.2 interactions. AlphaFold-Multimer provided novel insights into the functional roles of previously uncharacterized proteins in complexes. Our web portal (www.yeast-interactome.org) enables extensive exploration of the interactome dataset.

## Abstract (web summary)

A protein interaction network constructed with data from high-throughput affinity enrichment coupled to mass spectrometry provides a highly saturated yeast interactome with 31,004 interactions, including low-abundance complexes, membrane protein complexes and non-taggable protein complexes.

## Main

**Fig. 1:**
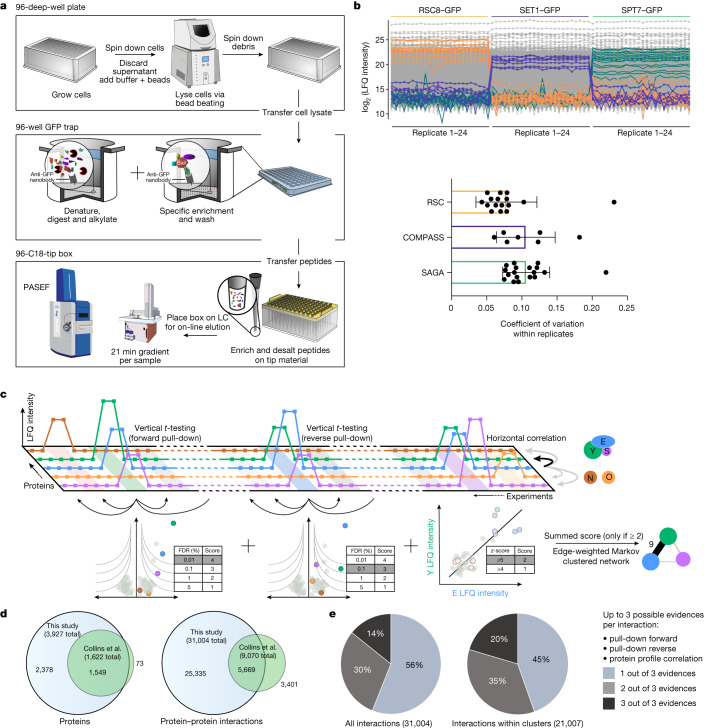
A comprehensive and scalable interactomics technology. **a**, Sample preparation in 96-well format and mass spectrometric measurement. Each strain of the GFP-tagged library is lysed by mechanical disruption and transferred to anti-GFP nanobody coated microtitre plates, where weak interactions are preserved by gentle washing. After enzymatic in-well digestion, resulting peptides are transferred to standardized C_18_-StageTips from which they are eluted directly into a standardized 60 samples per day gradient. Data are acquired in the PASEF scan mode on a trapped ion mobility—time-of-flight mass spectrometer. LC, liquid chromatography. **b**, Streamlined workflow and reduced transfer steps reduce the risk of manual errors and sample variation. Demonstration of workflow reproducibility and sensitivity on three nuclear complexes in biological replicates. A tagged member (bait) of each complex pulls down the known preys in very similar amounts based on label-free quantification (LFQ) intensities. Bottom, coefficient of variation (within 24 replicates), mean with standard deviation (*n* = 16 (RSC), 7 (COMPASS) and 18 (SAGA) complex members). **c**, Two-dimensional interaction scoring. Columns represent pull-down experiments in replicates (light colour). Squares depict intensities of detected proteins across the pull-down experiments. Three levels of evidence support each interaction: *t*-test of forward pull-down against complementary experiments, *t*-test of reverse pull-down, and protein profile correlation—the correlated abundance profile against all other proteins across all experiments (*z*-scored; Methods, ‘Protein correlation’). **d**, Overlap of proteins with at least one interactor and interactions detected in this study with the previous state-of-the-art network^2^. **e**, The proportion of interactions backed by multiple layers of evidence in the complete network and the network excluding inter-cluster interactions.

The large-scale study of cellular interactomes using mass spectrometry-based proteomics dates back over 20 years^3,4^, culminating in 2 studies in which nearly half the expressed yeast proteome was successfully purified with identified interactors^5,6^. These datasets have been mined extensively, leading to a network-based view of the cellular proteome. Given the importance of the interactome for functional understanding and the substantial improvements in mass spectrometry technology during the past decade^7,8^, we set out to generate a substantially complete interactome of all proteins present in an organism in a given state. We made use of an endogenously GFP-tagged yeast library containing the 4,159 proteins that are detectable by fluorescence under standard growth conditions^1^. Miniaturization and standardization of the workflow in combination with an ultra-robust liquid chromatography system with minimal overhead time coupled to a sensitive trapped ion mobility mass spectrometer utilizing the PASEF scan mode^9,10^ resulted in very high data completeness across pull-downs. This workflow required only 1.5 ml instead of litres of yeast culture, provided a constant throughput of 60 pull-downs per day and enabled the use of the same conditions for soluble or membrane proteins of vastly different abundances (Fig. 1a).

## Measurement of the yeast interactome

To test the quantitative reproducibility of our workflow, we performed 24 biological replicates of pull-downs of 3 nuclear complexes, which resulted in complete retrieval of these complexes from a single bait each, with 9% average coefficients of variation of enriched complex members (Fig. 1b). This compares with a 69% repeatability of assigned interactions in the previous large-scale screens^11^.

Three layers of evidence help to establish an interaction between two proteins. The first two are statistically significant enrichment of the proteins in the forward and in the reverse pull-downs (in which the prey pull-down significantly enriches the bait). Instead of using only a *t*-test of bait pull-down against a pull-down of a strain expressing GFP, we made use of our vast number of diverse GFP-tagged strains, to combine them into a single control group, thereby efficiently removing false positives not specifically binding to the bait (Methods, ‘Enrichment analysis’). Using this affinity enrichment (rather than affinity purification) concept^12^, we quantitatively compared all proteins across more than 8,000 pull-down measurements, making use of the profile similarities of interacting proteins in correlation analysis. This third type of evidence was highly informative owing to the high quantitative accuracy combined with a nearly complete set of virtual controls (Fig. 1c and Methods, ‘Protein correlation’).

We combined all three layers of each interaction into a single interaction score and retained those with a minimum score of 2, corresponding to: (1) a single pull-down at 1% false discovery rate (FDR); (2) a correlation *z*-score of at least 5; (3) forward and reverse pull-downs at 5% FDR each; or (4) a pull-down at 5% FDR combined with a correlation *z*-score greater than 4. To retrieve clusters and complexes from our interactome data, we used Markov clustering with the interaction scores as the edge weights, without any training or a priori knowledge (Fig. 1c and Methods, ‘Network generation’).

**Extended Data Fig. 1:**
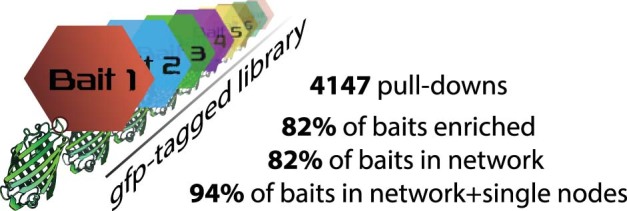
Schematic of the GFP-tagged library. 4,147 different endogenous c-terminally tagged yeast strains^1^ were used for 4,147 independent pull-down experiments. Each strain therefore allows the purification of the individually tagged protein (bait) and its specific interactors. The original library of 4,159 strains was reduced by twelve strains to 4147, due to updates in ORF annotations (see Methods: Data processing and normalization).

**Extended Data Fig. 2:**
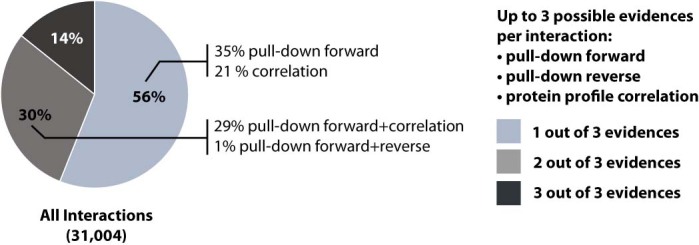
Detailed layers of evidence for all interactions. Detailed proportion of interactions backed by multiple layers of evidence.

The replicate GFP pull-down measurement in the 4,147 yeast strains resulted in the enrichment of 82% of the baits (Extended Data Fig. 1). Our mass spectrometry data provided statistically significant evidence for more than 30,000 physical interactions, corresponding to an average of 15.8 interactions per protein. Most were supported by forward pull-down (35%), followed by forward pull-down and significant prey correlation (29%), whereas nearly all interactions with both forward and reverse evidence also had significant correlation *z*-scores (95%) (Extended Data Fig. 2).

Owing to the limited overlap of the interactions reported by two previous large-scale studies (13% shared interactions), Collins et al. merged and reanalysed these datasets to create a consensus network with 1,622 proteins^2^ (nodes in a network). Our data encompass 95% of these proteins, but places nearly the entire expressed yeast proteome in a network (3,927 nodes). Our dataset of 30,000 significant protein–protein interactions confirms 63% of the much smaller Collins et al. dataset^2^ (Fig. 1d). Based on a comparison with the BioGRID database^13^, more than two-thirds of the interactions reported here are novel.

**Extended Data Fig. 3:**
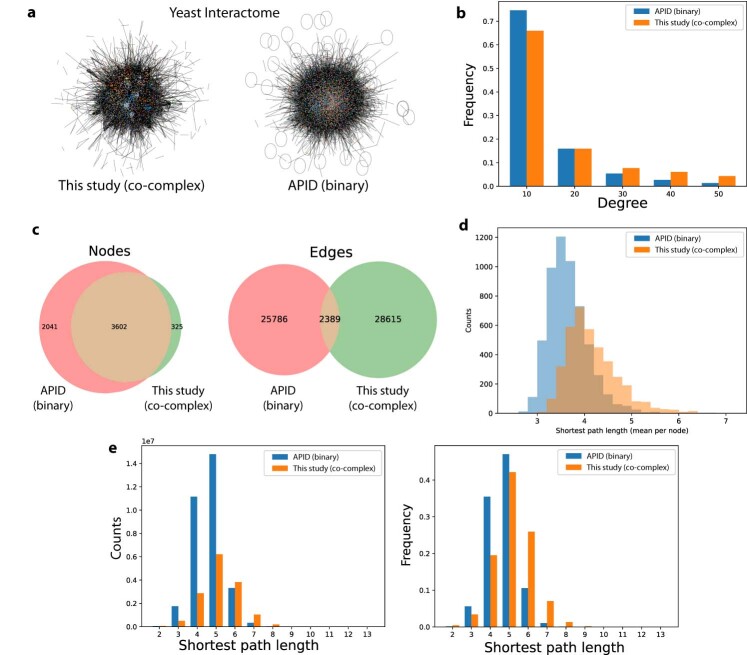
Comparison of affinity-enrichment purification coupled to mass-spectrometry (AE-MS) network (co-complex, this study) to curated binary network (from APID database). **a**, Edge weighted network (score and publication count, respectively) using spring-layout. Colors representing different Markov clusters. (Fruchterman-Reingold algorithm). **b**, Frequency of degree (number of interactors) of both datasets. **c**, Overlap of nodes and edges between both datasets. **d,e**, Distribution of shortest path length (mean per node and all).

Affinity enrichment coupled to mass spectrometry (AE–MS) is a ‘co-complex’-oriented approach, in contrast to binary interaction mapping. AE–MS can define interactions between all complex members even if their interaction is not direct but bridged via other members. By contrast, binary methods detect direct physical interactions between protein pairs^14,15^. By its nature, a large-scale, reproducible co-complex-oriented approach will therefore generate higher numbers of interactions overall, and especially in large complexes. In our dataset, the inherently high ‘redundancy’ in combination with an efficient scoring turned out to be fundamental for a clustering that identifies functional units. As also observed in human interaction data^15^, about one-quarter of published interaction data within our co-complex network overlaps that of binary detection methods (Extended Data Fig. 3).

## Organization of interactions in clusters

**Fig. 2:**
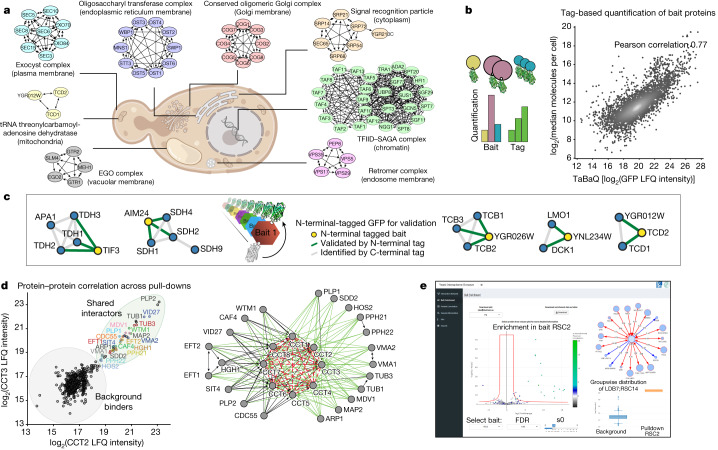
High-quality dataset for the exploration of the interactome. **a**, Clusters derived from our interactome for a range of challenging complexes such as chromatin-associated, soluble and membrane-bound complexes of various organelles. In each case, all known subunits were retrieved. The cell image was created with BioRender.com. **b**, Left, tag-based quantification enables retrieval of abundance information for the baits in a generic manner. Right, correlation of tag peptide-based signals with a literature compilation of yeast protein abundances^18^. **c**, Validation of selected clusters containing novel interactions or uncharacterized proteins using N-terminal GFP-tagged baits additional to the C-terminal tagged library used in the original screen. For the full validation panel, see Supplementary Fig. 1. **d**, For the non-taggable chaperonin containing t-complex (CCT), profile correlation analysis nevertheless reveals its subunits and interactors. Proteins with two and more interactions are shown. Interactions based on correlation only are shown in red (dashed) and previously unreported interactions with CCT are in green. **e**, The yeast-interactome web application (www.yeast-interactome.org) allows exploration of interaction data for interactions of interest. For all bait proteins, pull-downs are depicted as volcano plots together with a box plot that shows the mass spectrometry intensities of user-selected prey proteins. Subnetworks from pull-downs of the selected bait and reverse pull-downs or significant interactors can be displayed. Further features include full and subnetwork exploration, correlation visualization and an overview of sample quality.

Markov clustering analysis—with our interaction scores as edge weights—condensed the network into 617 clusters, with about 20,000 interactions, most supported by at least two statistically significant levels of evidence (Fig. 1e). When we inspected known protein complexes from different cellular compartments, especially membrane complexes, we found them to recapitulate the literature to a large degree. Furthermore, we retrieved 4,076 interactions between annotated membrane proteins, compared with 853 in a dedicated membrane proteome^16^. We show this exemplarily for the endosomal retromer complex, the conserved oligomeric Golgi complex and the plasma membrane exocyst complex, which are fully retrieved in our experiments (Fig. 2a). Our unbiased and high coverage analysis also identified novel subunits with tight association to known complexes. For instance, we found three subunits of the essential endoplasmic reticulum membrane oligosaccharyl transferase (OST) complex—an integral component of the translocon—associated with α-1,2-mannosidase (Mns1; human homologue, MAN1B1), an enzyme that catalyses the endoplasmic reticulum glycoprotein trimming reaction which is required for endoplasmic reticulum-associated protein degradation (ERAD). This indicates that the enzymatic activity of N-linked oligosaccharide chain addition is physically connected to the removal of a terminal sugar, at least in one isoform of the OST complex. The slow enzymatic activity of Mns1 acts as a timer^17^ and we speculate that it co-translationally primes stalled or erroneous proteins directly at its site of translocation for ERAD.

Many biological complexes share members and these can be difficult to disentangle by clustering algorithms. We speculated that our highly quantitative data could nevertheless resolve these cases. Applying a network layout algorithm (Methods, ‘Network generation’) to members of the transcription factor TFIID and SAGA complexes separately reconstructed these complexes while correctly assigning shared members (Fig. 2a). At the global scale, we found that about two-thirds of all interactions connected members within clusters, whereas the remainder connected clusters to each other. For example, the cytoplasmatic signal recognition particle (SRP) is connected to another cluster containing the SRP receptor (SRP101–SRP102). The largest connected clusters were the small and large subunits of the ribosome, with 400 inter-complex connections.

Leveraging the common, endogenous GFP tag on more than 3,405 detected baits, we next investigated whether the mass spectrometry signal of the GFP peptides could be used to quantify each bait. Indeed, these intensities correlated well (*r* = 0.77) with a recent compilation of yeast protein abundances^18^ (Fig. 2b). This validates our interaction workflow and enables tag-based estimation of the relative abundances of proteins in a cluster, which is useful to determine their functional role^19^.

For further validation, we used strains with N-terminally tagged proteins instead of C-terminally tagged proteins for a subset of baits of special interest (highlighted in Figs. 4 and 5). All baits associated with the corresponding 13 clusters that were tested and 12 of them confirmed novel interactions or had uncharacterized proteins in the cluster (Fig. 2c and Supplementary Fig. 1).

For some proteins—for example, the members of the chaperonin containing t-complex (CCT)—tagging is not possible because it interferes with protein stability or function^20^. Based on highly significant correlations between profiles of the subunits, CCT was nevertheless fully recovered (Fig. 2d). Besides the 8 conserved, ring-forming members, we also detected a distinct set of 21 interacting proteins, about half of which were previously unreported. Two of these were catalytic subunits of protein phosphatase 2A, suggesting regulatory functions, and others, such as tubulin and actin-related proteins (Tub1, Tub3 and Arp1) were major known folding substrates. CCT may have a restricted or broad set of folding substrates^21^, and our results quantitatively support the former possibility.

The above examples only scratch the surface of the interesting biological leads contained in the data. To enable ready exploration of interactions of interest, we created a web portal (www.yeast-interactome.org), which supplies statistical evidence for protein–protein associations, and summarizes the resulting clusters (Fig. 2e).

## Network architecture of the interactome

**Fig. 3:**
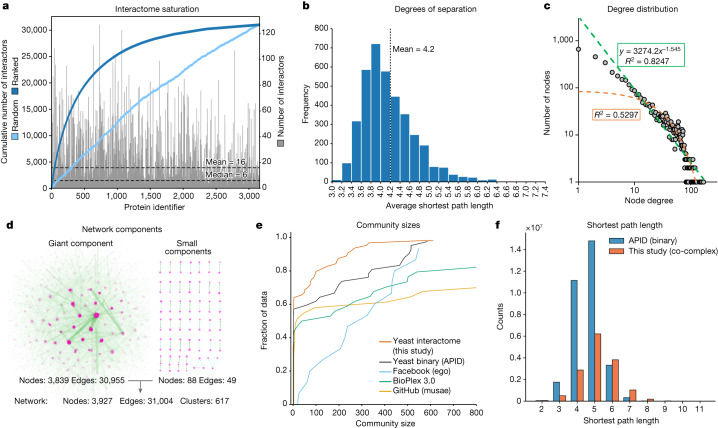
Properties of the protein interaction network. **a**, Distribution of the number of interactors. The sorted cumulative number of interactions reaches saturation at 30,000 interactions. **b**, The distribution of average shortest path length between all possible pairs of nodes within the giant component shows a mean of 4.2 steps, corresponding to 3.2 intermediaries (degrees of separation). **c**, Power law fit (green; equals a linear fit on a log–log scale) of the frequency of proteins with a given number of interactions highlights the scale-free properties of the network. An exponential fit is depicted in orange. **d**, Nearly all nodes of the network are connected with each other in the giant component. **e**, The cumulative distribution function of the community sizes (Louvain algorithm) detects more smaller communities for *S. cerevisiae* compared with other interactome datasets. **f**, Comparison of the yeast co-complex interactome (this study) to a curated yeast binary network (APID database^15^), showing the distribution of shortest path lengths.

**Extended Data Fig. 4:**
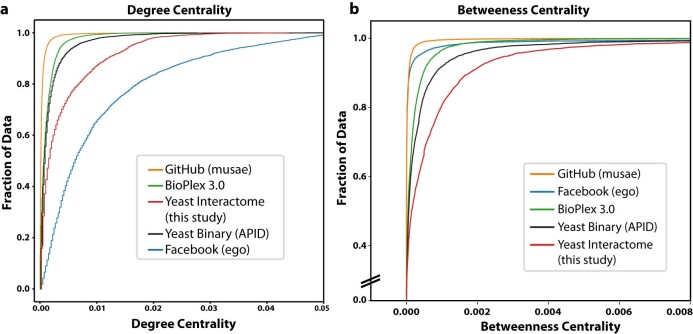
Comparison of centrality to different complex networks. **a**, Cumulative distribution function of the degree centrality. Comparison of different complex networks: *S. cerevisiae* has more influential (high degree centrality) nodes than BioPlex, APID and GitHub, and less than Facebook. **b**, Cumulative distribution function of betweenness centrality: The *S. cerevisiae* interactome has more nodes with a high betweenness-centrality than the comparison data sets.

The availability of data for large networks in systems including power grids, genetic networks and human social networks has enabled the study of their underlying architecture, commonalities and differences^22^. This topic also has a long history in protein interaction networks. However, these analyses have been limited by the incompleteness of the data, especially in multicellular species^23^. With an in-depth protein–protein interaction map in hand, we compared its characteristics with networks in different domains. Yeast proteins are highly connected, with an average of 16 and a median of 6 interactions per protein, significantly more than the human BioPlex interactome^24^ (average interactions: 8) (Fig. 3a). Influential nodes—those with the highest number of normalized interactors (or degree centrality)—were more common than in the yeast binary dataset and the human BioPlex interaction dataset (Extended Data Fig. 4a).

One of the key features of most real-life networks with complex topology in contrast to random networks is the scale-free power law distribution of interactors^25,26^. Scale-free network properties are thought to arise by preferential attachment over evolutionary time to already well-connected nodes and can be identified by a linear relation of the node degree or number of interactors with its frequency (number of nodes with that degree) plotted in log–log space. Although this has been difficult to demonstrate for biological networks (they instead appear to be exponential or have a truncated power law degree distribution^27^), our yeast interactome clearly indicates scale-free properties (Fig. 3c). In accordance with previous protein–protein interaction networks^5,28^, the exponent was below 2, at the lower end of the range 2 to 4 for other scale-free networks.

**Extended Data Fig. 5:**
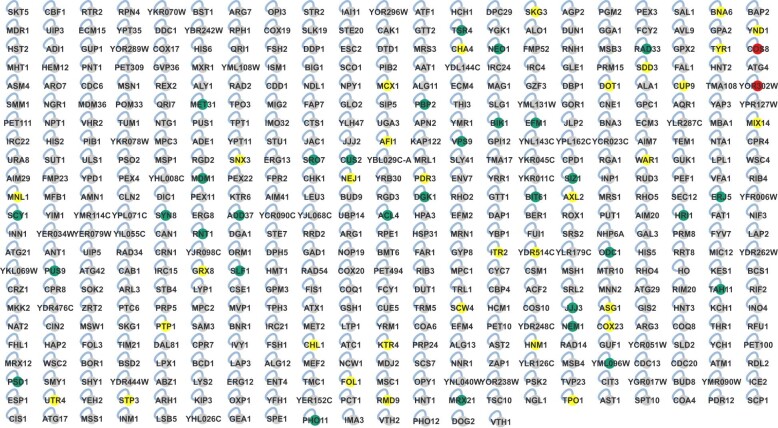
“Asocial” proteins. Representation of 476 significantly enriched and detected bait proteins that lack any significant interactor under given conditions in this study. Colored: Random selection of 65 baits for N-terminal GFP tagged pull-downs retrieved interactors for about have of them (yellow, no interactor; green, interactors; red, no bait detected). Blue edges depict self-edges.

The high connectivity of most proteins organizes almost all of them (3,839) into a single giant connected component, accompanied by 41 small components (88 proteins) (Fig. 3d). A total of 476 proteins were outside of the network because mass spectrometry analysis of their pull-downs only identified the bait itself. There was a highly significant enrichment for 94% of these baits (FDR < 0.01%), indicating that there were no identifiable interactors under our standard conditions despite a successful pull-down (Extended Data Fig. 5, see volcano plots on the web portal).

We next investigated the large-scale organization of the yeast interactome using the Louvain community detection algorithm (Methods, ‘Network comparisons’). This revealed that yeast is organized in smaller communities than the yeast binary curated network or the human Bioplex (Fig. 3e). Important ‘bottleneck’ proteins that form part of many shortest paths have a high ‘betweenness centrality’. The yeast interactome has comparably more of these central nodes, and bioinformatic enrichment analysis highlighted proteins involved in ‘RNA polymerase II’, ‘mitochondrial nucleoid’, ‘gluconeogenesis’ and ‘misfolded protein binding’ (Extended Data Fig. 4b and Extended Data Table 1).

Altogether, based on the total of 4,403 identified yeast proteins, with 74.1% having at least two interactors, 15.1% had one and only 10.8% had no discernable interaction partner. To investigate whether the latter set is truly ‘non-social’ or is an artefact of expression level or its tag position, we performed our workflow on a subset of the proteins using N-terminal tagged strains with identical promoters^29^ (Extended Data Fig. 5). This yielded additional interactors for about half of the proteins. Notably, the overall average of identified interactors in this set was around 2, compared with 16 in the main dataset, indicating that this set of proteins was indeed poorly connected (Supplementary Fig. 2). Although reciprocal tagging was beneficial, complexes with higher numbers of interactions would already be picked up by the redundancy effect of our screen. Given that some of our baits will have context-dependent interactions that are not captured here, our estimates are conservative and we conclude that almost all yeast proteins are ‘social’.

## Insights from global organization

Intensive research over the past decades has made *S. cerevisiae* arguably the best understood single-cell eukaryotic organism, leading to the discovery of crucial conserved cellular functions such as metabolic pathways, mechanisms of DNA replication and transcription, protein quality control and modifications that were later confirmed in human and other organisms. Nevertheless, our interactome contained uncharacterized proteins or interactions that are not reported in the BioGRID^13^ database and thus provides novel biological insights (extended selection in Supplementary Fig. 3). Furthermore, BioGRID has accumulated binding events from very disparate experiments without a common confidence score (133,900 physical interactions from about 10,000 publications). We reasoned that our homogeneous, high-quality dataset would help biologists to highlight true positive interactors with biological relevance, several of which we discuss below.

**Fig. 4:**
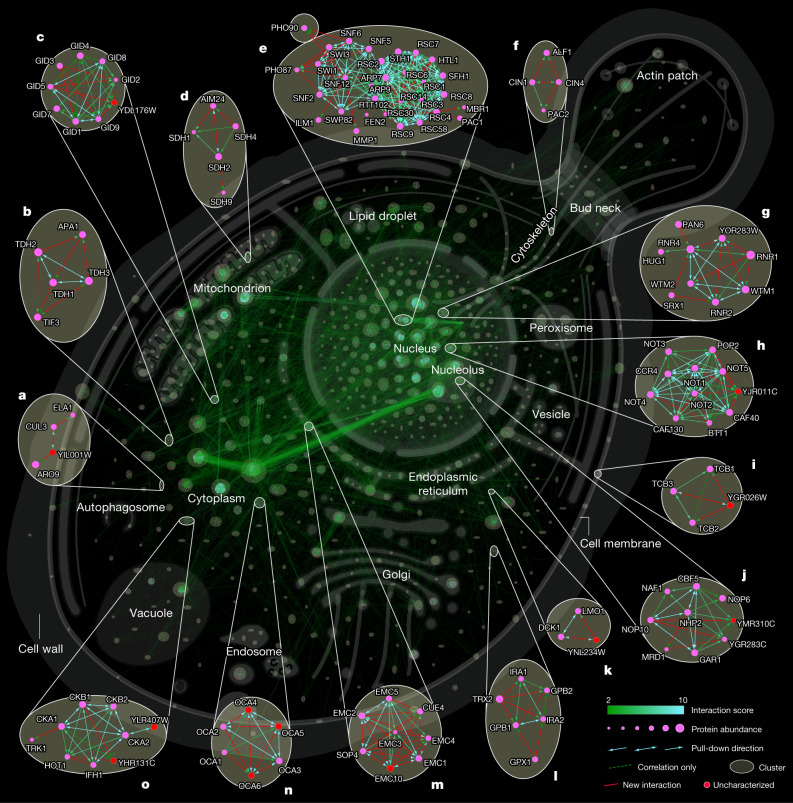
Network of an in-depth interactome, highlighting novel interactions. Cellular interaction map of all significant interactions. Clusters are highlighted by circles and cellular localization is indicated by most frequent gene ontology term (cellular component) within a cluster. **a**–**o**, Enlargements show examples of either novel interactions (based on BioGRID^13^) or those that have not been described further as potential high-significance interactors and interactions involving uncharacterized proteins. A full browsable and interactive (Cytoscape^56^) version of this network is available at www.yeast-interactome.org.

A total of 11 evidences connect the uncharacterized protein YDL176Wp with the conserved glucose-induced degradation (GID) complex, only a few of which had been indicated by previous pull-down or genetic interaction data^5,30^ (Fig. 4c). These types of high-confidence associations assist in prioritizing interactions and form the basis for a detailed mechanism and structure discovery of a novel GID modulator^31^. Similarly, our data ties the uncharacterized protein YJR011Cp to the conserved transcription and translation regulatory CCR4–Not complex^32^ via high-significance interactions to a majority of its subunits (Fig. 4h). Finally, YHR131Cp is linked to three subunits of the kinase CK2 and YLR407Wp is linked to the fourth subunit (Fig. 4o).

We identified an interaction of Cue4—a protein of unknown function containing a ubiquitin-binding domain—with the endoplasmic reticulum membrane complex (EMC), a potential membrane protein chaperone (Fig. 4m). As Cue4 is a paralogue of Cue1 (coupling ubiquitin conjugation to endoplasmic reticulum degradation), a component of ERAD^33^, this physical link and the known aggravating genetic interactions of *∆cue1* with EMC knockouts^34^ suggests an ERAD-related quality control mechanism for EMC.

The transcriptional regulator SWI/SNF unexpectedly interacts with the phosphate transporters Pho87 and Pho90 (Fig. 4e). Out of four plasma membrane phosphate transporters only Pho87 and Pho90 have a cytoplasmatic accessible SPX domain. Although an SPX-dependent phosphate-sensing mechanism has been found in plants^35^, such a mechanism remains unknown in *S. cerevisiae*. In *Arabidopsis*, inositol pyrophosphate (InsP_8_) concentration increases under phosphate-rich conditions and promotes the interaction between SPX domains and a four-stranded coiled-coil motif of phosphate starvation response transcription factors^36^. Notably, the recently solved structure of SWI/SNF reveals such a coiled-coil four-helix bundle at its spine region^37^, providing a potential SPX interaction site. This raises the possibility of a novel cytoplasmatic sensing and retention mechanism of this key transcriptional regulator, which is known to be necessary for a phosphate starvation response^38,39^. Of note, both the SWI/SNF complex and a SPX domain-containing phosphate transporter (XPR1, which has recently been shown to be controlled by InsP_8_^40^) are present in humans.

Illustrating translational relevance, we expand the known interaction of the GTPase-activating proteins Ira1 and Ira2 (neurofibromin (NF1) in humans), and Gpb1 and Gpb2 (ETEA in humans)^41^ by Trx2 a thioredoxin isoenzyme (human homologue, TXN) and Gpx1 (human homologues, GPX3–6), an antioxidant enzyme whose glutathione peroxidase activity is neuroprotective in models of Huntington’s disease^42^ (Fig. 4l).

Additionally, we find a new physical interaction between the two uncharacterized proteins YPR063Cp and YNR021Wp (Supplementary Fig. 3), whose dimerization and structure has just been predicted in a deep learning approach^43^.

As well as known and novel protein complexes, the yeast interactome (depicted in Fig. 4) clearly shows evidence of high order connections. These often map to different compartments of the cell, such as the prominent connections between ribosomes in the cytoplasm and the nucleolus, its site of maturation or those that connect large and small ribosomal subunits that despite their ‘stickiness’ are organized in individual clusters.

## Structural prediction by deep learning

**Fig. 5:**
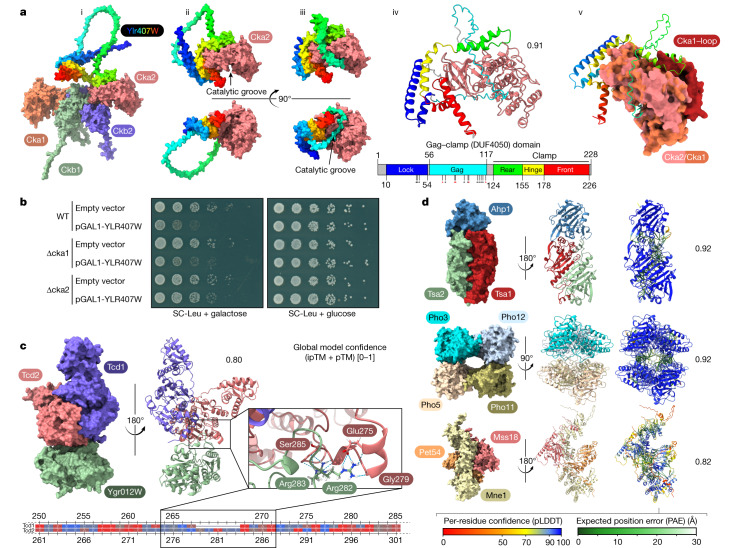
Interaction-based structure prediction. **a**, Structure and binding mode of YLR407Wp to Cka2 as modelled in AlphaFold-Multimer. (i) Competitive binding mode of the YLR407Wp front clamp binding domain with the CK2 holoenzyme. (ii) and (iii) Two major predicted models with the unstructured loop acting either as a lasso or a catalytic groove gag. (iv) Motifs of the conserved DUF4050 domain are coloured and named based on the modelled structure. Annotated phosphorylation sites are indicated with asterisks (black, reported twice in UniProt; red, reported three or more times). (v) Superposition of Cka1 and Cka2, with the clash of the Cka1-specific insertion loop with the rear-clamp domain highlighted. **b**, Serial dilution spotting of yeast strains carrying a Leu marker plasmid overexpressing YLR407W under the galactose inducible promoter or a negative control (empty vector). The growth defect phenotype caused by YLR407W overexpression is dependent on the presence of the interactor Cka2 but not on the non-interactor Cka1. **c**, Suggested binding mode of the two homologous proteins Tcd1 and Tcd2 with YGR012Wp. The alignment is coloured based on amino acid side chain hydrophobicity, with the highest in red to the lowest in blue. pTM, predicted template modeling score; ipTM, interface pTM. **d**, Selection of high-confidence complexes and their scoring (for an extended version see Supplementary Fig. 4).

We envisioned that recent breakthroughs in protein structure prediction^44–46^, including the ability to model several subunits of protein complexes^43,47^, would add an additional dimension to our interactome dataset. Considering the current computational limitations for larger protein complexes (Methods, ‘Structure predictions’), we started with all clusters in our interactome that contained uncharacterized proteins or unreported interactions (Supplementary Fig. 3). We ran Alphafold2 on all of these below a size limit (2,500 amino acids) and recorded results on those that yielded a model confidence score of at least 0.7. For increased coverage, we then refined the results by running AlphaFold2 on a subset of clusters of interest. From the entire collection of these structurally predicted complexes (Supplementary Fig. 4), we focused on a selection with high confidence score and biological interest (Fig. 5).

CK2 is an essential eukaryotic kinase that is involved in a multitude of cellular pathways covering most hallmarks of cancer—such as cell death evasion and promotion of cell proliferation. The holoenzyme is built from two regulatory beta and two catalytic active alpha subunits^48^ (Ckb1, Ckb2, Cka1 and Cka2). Our cluster for the yeast CK2 contains the uncharacterized interacting protein YLR407Wp, and CK2 was its only interaction partner (Fig. 4o). Furthermore, this interaction with Cka2 is direct and does not include the other subunits (and vice versa, validated by N-terminal tagging; Supplementary Fig. 1), suggesting a competitive binding mode. This was supported by the predicted structure, in which one of the two binding domains of YLR407Wp directly overlaps with the Ckb1–Ckb2-binding interface of Cka2 (Fig. 5a (i)). YLR407Wp contains a conserved domain of unknown function (DUF4050) that is found in more than 3,000 proteins across 1,000 eukaryotic species according to InterPro^49^. The structural models revealed that only the bound state has structurally defined domains, namely a clamp region (rear-hinge-front) and a lock region that closes the structure by binding to the front clamp (Fig. 5a (iv)). Modelling suggested two conformations, one in which a prominent loop is unstructured and reminiscent of a lasso and one in which it fits like a ‘gag’ into the catalytic groove (Fig. 5a (ii) and (iii)). Notably, UniProt annotations indicate that this region contains up to 12 phosphorylatable sites, suggesting a kinase-driven mechanism of conformation change.

For this reason, we renamed YLR407Wp to Gag1. The major structural difference between the two similar catalytic subunits Cka1 and Cka2 is an insertion loop at the rear of Cka1 (S91–N128). This loop clashes with the potential binding of Gag1 (Fig. 5a (v)), which would explain Cka2 selectivity and provide a functional reason for this structural difference. Phenotypic assays support this competitive and selective binding mode, which is already based on strong interaction and modelling data as well. The growth defect caused by overexpression of YLR407Wp can be rescued by the deletion of the interacting subunit Cka2, but not by the deletion of the non-interacting subunit Cka1 (Fig. 5b).

**Extended Data Fig. 6:**
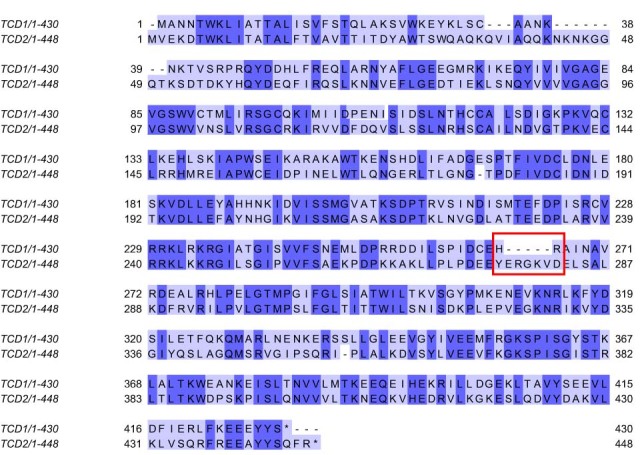
Sequence alignment of Tcd1 and Tcd2. Alignment of the paralogs Tcd1 and Tcd2 highlighting the insertion loop (red square).

We also identified a novel complex defined by three unreported interactions (all with the maximum interaction score of 10) between Tcd1, Tcd2 (mitochondrial proteins that are involved in tRNA base modification) and YGR012Wp (a protein of unknown function that we rename to Tcd3) (Figs. 2a and 5c). A homologue of Tcd1 and Tcd2 in *Escherichia coli* (TcdA) functions in a complex of three proteins in the cyclization of an essential tRNA modification that is found in all three domains of life, including humans^50^. Whereas the two paralogues Tcd1 and Tcd2 build the main interaction interface, Tcd3 interacts largely with Tcd2. Our modelling suggests that this interaction is enabled by a characteristic insertion loop (amino acids 277–281) of Tcd2 that is not present in Tcd1 (see alignment in Fig. 5c and Extended Data Fig. 6), thereby explaining the preferential interaction of Tcd3 with Tcd2 over Tcd1.

A third example shows how structural information based on interaction data helps to foster biology-driven hypotheses. In our model of the newly discovered interaction of ubiquitin E3 scaffold protein Cul3 with YIL001Wp (human homologue, ABTB1), Cul3 binds at the location where known adapter proteins are located (Fig. 4a and Supplementary Fig. 4g).

## Outlook

Here we have developed and applied a novel and highly scalable interactome technology, enabling replicate measurement of the yeast network in a fraction of the measurement time and starting materials needed previously. Our screen reached near saturation and contained nearly all complexes that were expected under our experimental conditions (Figs. 3a and 4). We show that high-confidence interaction data provides an ideal foundation for recently developed deep learning models that predict complex structures from their sequences^43–45,47^, resulting in functionally relevant de novo structural models.

The high connectivity of the resulting network is reflected in a mean shortest path between yeast proteins of 4.2, ranging from highly connected proteins with only three steps to less connected ones with an average of more than 7 steps (Fig. 3b). This is very similar to the 4.7 path length for world-scale Facebook relationships^51^.

Given its streamlined nature, our workflow can now be readily used in other endogenously tagged model organisms^52^ or to study remodelling of the interactome in the presence of dynamic biological processes or perturbations. Similarly, we envision its use with other interaction technologies such as BioID or APEX^53^ using tagged libraries that can be easily generated using platforms such as SWAp-Tag^54^. The comprehensive yeast interactome data can be further used as prior knowledge for hypothesis-driven analysis of protein complexes—for example, for native protein complex co-fractionation coupled to mass spectrometry^55^. Additionally, we imagine that such interactome data could also be combined with mass spectrometry–crosslinking studies.

## Methods

### Cell growth

To achieve samples with similar cell numbers, pre-cultures of the *S. cerevisiae* GFP-tagged library^1,29^ were grown in YPD medium (1% yeast extract, 2% bacto peptone, 2% glucose) for two days in 2 ml, U-bottom 96-deep-well plates. This allowed cell concentration convergence of different strains during the slow growing post-exponential phase. Cells were resuspended and 50 µl of each pre-culture was used to inoculate 1.5 ml of fresh YPD medium (corresponding to an optical density of 0.5 at 600 nm) in 96-deep-well plates (LoBind, 2 ml, 0030504305, Eppendorf). Plates were covered with an air-permeable membrane and incubated while shaking at 300 rpm and 30 °C for 6 h. This allowed the progression through the lag phase and three cell cycles followed by collection under standard growth conditions. Cells were pelleted in the 96-deep-well plates by centrifugation at 3,500 rpm (2,451*g*) for 5 min. The supernatant was discarded by fast decanting and quick dabbing on paper towels. Plates with pellets were sealed with plastic covers and stored at −80 °C until cell lysis.

### Cell lysis

Deep-well plates with cell pellets were thawed on ice for 5 min. 100 µl of glass beads (0.5 mm, acid-washed, G8772, Merck) were added to each well using a 96-well bead dispenser (LabTIE International). After 5 min 250 µl of 4 °C cold lysis buffer (50 mM Tris HCl pH 7.5, 150 mM NaCl, 5% glycerol, 0.05% IGEPAL CA-630, protease inhibitor EDTA-free (cOmplete, 1 tablet per 50 ml, 11873580001, Merck), 1 mM MgCl_2_, 0.75 U µl^−1^ in-house *Serratia marcescens* endonuclease/SmDNase) were added. Plates were sealed using a heat sealer (S200, 5392000005, Eppendorf), the low-profile plate adapter (5392070020, Eppendorf) and transparent heat-sealing films (0030127838, Eppendorf) for 2 s at 180 °C and immediately put back on ice. Cell lysis was performed within the 96-deep-well plates at 4 °C via bead-beating (2010 Geno/Grinder, SPEX SamplePrep) for 4 cycles of 1.5 min each at 1,750 rpm. Plates were cooled in ice water and covered with ice for 7 min in between cycles and for 10 min after the last cycle. 4 plates were processed in parallel during bead-beating and top and bottom positions were switched at each cycle. Cell debris was spun down at max speed (4,300 rpm (4,347*g*)) for 10 min at 4 °C. Plates were carefully put back on ice and immediately used for the pull-down protocol (Fig. 1a).

### Interactor enrichment

Pull-downs and all sample handling steps were performed at 4 °C. Anti-GFP nanobody coated 96-well microtitre plates were custom made and optimized for this protocol allowing efficient and high reproducible in-well digestion, and mass spectrometry compatibility (plates are now commercially available as GFP-Trap Multiwell Plate, gtp-96, Chromotek). Plates were prepared with 200 µl wash buffer 1 (50 mM Tris HCl pH 7.5, 150 mM NaCl, 5% glycerol, 0.05% IGEPAL CA-630) per well on a shaker for 1 min at 800 rpm followed by removal of the buffer. The cell lysates were carefully transferred from the 96-deep-well plates by slow uptake of 175 µl supernatant without dislodging glass beads nor the cell debris pellet to the GFP-Trap plate. The GFP-Trap plate was incubated for 1 h at 800 rpm on a small stroke (3 mm) shaker (TiMix 5 control, Edmund Bühler) to enrich for GFP-tagged proteins and their interactors. Cell lysates were discarded and plate wells were washed twice with 200 µl wash buffer 1 and twice with wash buffer 2 (50 mM Tris HCl pH 7.5, 150 mM NaCl, 5% glycerol). To allow stable binding of unspecific background proteins—an important factor for label-free quantification—wash buffer was added slowly, and plates were not shaken during wash steps. Emptied, protein-enriched plates were covered and stored at −80 °C until mass spectrometry sample preparation (Fig. 1a).

### Sample preparation for mass spectrometry

Protein-enriched GFP-Trap plates were brought to room temperature and 50 µl of digestion mix 1 (4.5 M urea, 1.5 M thiourea, 10 mM Tris HCl pH 8.5, 3 mM dithiothreitol, 2 ng µl^−1^ LysC) were added per well. Plates were incubated at 30 °C and 1000 rpm on a small stroke (3 mm) shaker. After 3 h, 100 µl of digestion mix 2 (10 mM Tris HCl pH 8.5, 7.5 mM chloroacetamide, 2 ng µl^−1^ LysC) were added and microtitre plates and lids were sealed with parafilm. The plates were incubated overnight at 30 °C at 800 rpm. The reaction was stopped and the sample was acidified with 15 µl of 10% TFA per well. Plates with peptides were stored at −80 °C till sample loading on EvoTips (Evosep) (Fig. 1a).

### Loading of peptide samples on Evotips

Evotips (Evosep) were activated for 5 min in a 1-propanol Evotips-box reservoir at room temperature, followed by a wash step with 50 µl buffer B (acetonitrile with 0.1% formic acid) and centrifugation at 500*g* for 1 min at room temperature. The flow-through was discarded and Evotips were placed back into 1-propanol. Evotips were conditioned with 50 µl of buffer A (ddH_2_O with 0.1 % formic acid) and centrifugation at 500*g* for 1.5 min at room temperature and were placed in a container with buffer A. Forty microlitres of thawed peptide sample were loaded and Evotips were centrifuged at 500*g* for 1.5 min at room temperature and placed back in a container with buffer A. Two-hundred microlitres of buffer A was added and partially washed through the Evotips by centrifugation at 500*g* for 50 s. Evotips boxes with buffer A at the container bottom were placed on the Evosep One liquid chromatography platform (Evosep, Odense, Denmark) for liquid chromatography–mass spectrometry (LC–MS) analysis. Pull-downs were acquired in technical duplicates and the injection order was reversed after the first measurement (Fig. 1a).

### Liquid chromatography

For separating peptides by hydrophobicity and eluting them into the mass spectrometer, we used the EvoSep One liquid chromatography system and analysed the yeast interactome pull-down proteomes with the standardized 21 min (60 samples per day) gradient. We employed a 15 cm × 150 μm inner diameter column with 1.9 μm C18 beads (PepSep) heated at 60 °C coupled to a 20 µm ID electrospray emitter (Bruker Daltonik). The column was replaced between replicate measurements. Mobile phases A and B were 0.1 % formic acid in water and 0.1 % formic acid in acetonitrile, respectively. The EvoSep system was coupled online to a trapped ion mobility spectrometry quadrupole time-of-flight mass spectrometer^57^ (timsTOF Pro, Bruker Daltonik) via a nano-electrospray ion source (Captive spray, Bruker Daltonik). A 24-fraction library of wild-type *S. cerevisiae* was generated using the high-pH reversed-phase ‘spider fractionator’^58^ and data were acquired using the same sample set-up.

### Mass spectrometry

Mass spectrometric analysis was performed in a data-dependent (dda) PASEF mode. For ddaPASEF, 1 MS1 survey trapped ion mobility spectrometry–mass spectrometry (TIMS–MS) and 4 PASEF tandem mass spectrometry (MS/MS) scans were acquired per acquisition cycle. The cycle overlap for precursor scheduling was set to 2. Ion accumulation and ramp time in the dual TIMS analyser was set to 100 ms each and we analysed the ion mobility range from 1/*K*_0_ = 1.3 V s cm^−2^ to 0.8 V s cm^−2^. Precursor ions for MS/MS analysis were isolated with a 2 Th window for *m*/*z* < 700 and 3 Th for *m*/*z* > 700 in a total *m*/*z* range of 100–1,700 by synchronizing quadrupole switching events with the precursor elution profile from the TIMS device. The collision energy was lowered linearly as a function of increasing mobility starting from 59 eV at 1/*K*_0_ = 1.6 V s cm^−2^ to 20 eV at 1/*K*_0_ = 0.6 V s cm^−2^. Singly charged precursor ions were excluded with a polygon filter (otof control, Bruker Daltonik). Precursors for MS/MS were picked at an intensity threshold of 2,000 arbitrary units (a.u.) and re-sequenced until reaching a target value of 24,000 a.u. considering a dynamic exclusion of 40 s elution. The capillary voltage was set to 1,600 V and dry gas temperature to 180 °C.

### Raw data processing

Mass spectrometry raw files were processed using MaxQuant (v1.6.17.0)^59,60^, which extracts features from four-dimensional isotope patterns and associated MS/MS spectra, on a computing cluster (SUSE Linux Enterprise Server 15 SP2) utilizing UltraQuant (github.com/kentsisresearchgroup/UltraQuant). To allow processing in an acceptable time frame, RAW files were handled in 5 parallel batches of approximately 1,700 files each containing plates equally distributed across the measurement period. Files were searched against the *S. cerevisiae* Uniprot databases (UP000002311_559292; canonical and isoform, reviewed-sp and unreviewed-tr from 02/2020). For high-significance identification the FDRs were reduced and controlled at 0.1% both on peptide spectral match and protein levels. Peptides with a minimum length of seven amino acids were considered for the search including N-terminal acetylation and methionine oxidation as variable modifications and cysteine carbamidomethylation as fixed modification, while limiting the maximum peptide mass to 4,800 Da. Enzyme specificity was set to LysC cleaving C-terminal to lysine. A maximum of two missed cleavages were allowed. The parameter ‘type’ was set to ‘TIMS-DDA’ with ‘TIMS half width’ at 4. The instrument was set to ‘Bruker TIMS’ and main search peptide tolerance reduced to 8 ppm, the maximum charge set to 5 and minimum peak length to 3. Peptide identifications by MS/MS were transferred by matching four-dimensional isotope patterns between the runs (4D-MBR) using a narrow elution match time window of 12 s and a reduced ion mobility window of 0.01 1/*K*_0_. Protein quantification was performed by label-free quantification using a minimum ratio count of 2. The 24-fraction library was added as an additional parameter group with the same group-specific settings, but LFQ disabled and ‘separate LFQ in parameter groups’ under global parameters enabled. The writing of additional tables was disabled for performance reasons.

### Data processing and normalization

Twelve outdated samples of the GFP library were eliminated. These included wrongly annotated open reading frames that were merged with others: YAR044W, YPR090W, YDR474C, YFR024C, YJL021C, YJL017W, YGL046W, YFL006W, YGR272C, YBR100W, YJL018W and YJL012C-A. After the removal of potential contaminants, reverse and ‘only identified by site’ hits, MaxQuant proteinGroups.txt output files from the five batches were merged using the majority protein IDs column. Values were filtered for two valid values within at least one replicate group. To adjust for potential differences between the 5 MaxQuant batches caused by the parallel applied label-free normalization algorithm and for potential handling batch effects between 96-well plates, values were median-normalized if there were more than 5% of valid values in each of the corresponding groups.

### Missing value imputation

Missing values were imputed in a two-tiered approach. For proteins with measured values in more than 5% of all samples (or minimally 400 samples), a protein-specific missing value imputation approach was used. Here, a random value was sampled from a normal distribution with following properties: mean = median of all measured intensity values for the given protein, standard deviation = standard deviation of all measured intensity values for the given protein. Lower and upper bounds for the normal distribution were set to three standard deviations from the mean and minimally to zero. The function rtruncnorm from the R library truncnorm was employed. For proteins with less than 5% valid values (or in less than 400 samples), global metrics were employed for missing value imputation. Here, missing values were sampled from a normal distribution with the following parameters: mean = mean of all quantified values across all proteins and samples minus 1.8 times the standard deviation, standard deviation = the standard deviation of all quantified values across all proteins and samples multiplied by 0.3. The accompanying R script is in Supplementary Data 1 as Preprocessing.R.

### Protein correlation

Due to the large sample number that would negatively influence correlation, we chose a subsampling approach: For each protein pair across the sample profile, the top 2% of samples with the highest intensities for both proteins were selected (resulting in 2–4% depending on their overlap) and complemented by twice the number of randomly selected samples as background. The selected subset of samples was used to calculate the Pearson correlation coefficients of the protein pair (Fig. 1c). The effect of weighted correlation can be visualized by enabling ‘subsample values’ under protein correlation in our web application (yeast-interactome.org). Since the distributions of correlation coefficients varies between proteins and in order to define a universal cut-off for significant correlations, correlation coefficients were normalized via row wise *z*-scoring. A *z*-scored Pearson correlation coefficient above 4 and 5 therefore corresponds to a chance probability of below 3.2 × 10^−5^ and 2.9 × 10^−7^, respectively. The accompanying Python script is available in Supplementary Data 1 as CorrelationAnalysis.py.

### Enrichment analysis

A two-tailed Welch’s *t*-test was performed on each replicate-grouped pull-down sample using all corresponding complement samples as a combined control^12^. Within the combined control group, samples with the highest bait correlation (top 5%) were excluded in order to provide a bait-unrelated control. FDR cut-off lines were calculated using an analytical approach using an *S*_0_ parameter of 0.5 (ref. ^61^). The accompanying R script is available in Supplementary Data 1 as DifferentialAnalysis.R. We performed analysis for the N-terminal subset using PerseusNet^62^.

### Network generation

Interactions for the first two layers of evidence (forward and reverse pull-down) were defined between bait proteins and significantly enriched prey proteins from the *t*-tests. They were scored based on their FDR of 5%, 1%, 0.1% and 0.01% at 1, 2, 3 and 4, respectively (score_FDR). For the third layer of evidence, an interaction for *z*-scored Pearson correlation coefficients above 4 and 5 was scored at 1 and 2, respectively (score_cor). All three layers of evidence were combined into a single interaction score ranging from 1–10 (score_FDR+cor), thereby weighting interactions based on their experimental significance (Fig. 1c). The accompanying R script is available in Supplementary Data 1 as networkCreatoR.R. Networks were created and exported into Cytoscape^56^ for further analysis and visualization strategies. The network was filtered for interactions with a combined score equal to or above 2, thereby excluding interactions based only on a single *t*-test with an FDR of above 1% or a *z*-scored Pearson correlation coefficient of below 5. Further individual filtering can be achieved via the edge columns (that is, scores) within the Cytoscape filtering tab or any other table handling software. The Markov clustering algorithm was applied using the interaction score as edge weight and a granularity parameter of 2.5 while retaining inter-cluster edges (interactions). Clustering calculations were performed with 16 iterations and not stopped if residual increased. The CompoundSpringEmbedder (CoSE) layout algorithm was applied to single clusters using an ‘ideal edge length’ parameter of 150. A small subset of baits that did not generate a significant number of mass spectrometry detectable peptides, but still enriched significantly for preys are marked as ‘inferred from bait’. Protein abundance is based on the intensity of the GFP tag in each sample (tag-based quantification, indicated in the TaBaQ column; see Fig. 2b) and the relative size of the nodes in the network is based on that value. The network including edges (interactions) and nodes (proteins), annotations, scores, and layouts (including the ‘highlight novel’ style) can be downloaded as Cytoscape session file at (www.yeast-interactome.org). Alternatively, the Cytoscape session file and the interaction data including annotations and scores can be found in Supplementary Data 2 as The_Yeast_Interactome.cys, The_Yeast_Interactome_Edges.csv and The_Yeast_Interactome_Nodes.csv.

### Organelle-based mapping of clusters

The AutoAnnotate plugin^63^ v1.3.5 was used to generate a single localization-based term for each Markov cluster utilizing WordCloud^64^. Within WordCloud clustering and normalization was disabled and AutoAnnotate was run using a ‘minimum word occurrence’ and ‘max. words per label’ of 1. Therefore, based on the Uniprot localization annotation (most abundant word within the ‘Subcellular localization [CC]_simplified’ column), a single cellular localization term was defined for most clusters. Within the Cytoscape group preferences the attribute aggregation was enabled and the ‘visualization for group’ was set to ‘none’. Collapsed localization (collapse singleton clusters enabled)-based labelled groups were organized using the ‘Boundary layout’ using self-defined areas representing major cellular organelles. Node repulsion was increased to 1,000,000. Clusters were expanded and their positions manually adjusted. For cluster annotation the standard complex name from EMBL Complexportal was used. For each cluster the most frequent names is shown, (maximum words per label: 1, minimum word occurrence: 2; normalization and clustering off). An extended naming can manually be selected in Cytoscape under AutoAnnotate labelled ‘Complex name (long)’. The image of the background cell in Fig. 4, the Cytoscape session and the web application is an adopted version from SwissBioPics by the Swiss-Prot group of the SIB Swiss Institute of Bioinformatics^65^.

### Network comparisons

Network comparison analysis was performed in Python 3.8.1. Tabular data was loaded via the pandas package (1.3.1) and converted to a network via NetworkX (2.6.2). To calculate ‘Betweeness’ and ‘Degree centrality’, the respective NetworkX functions were used. To perform community analysis, a Python implementation of the Louvain algorithm was used (https://github.com/taynaud/python-louvain, version 0.15). Cumulative distribution functions were plotted using the matplotlib-library (3.4.2) and NumPy (1.20.3). Reference datasets were downloaded from the Stanford Large Network Dataset Collection (http://snap.stanford.edu/data/), the BioPlex Interactome homepage (https://bioplex.hms.harvard.edu/interactions.php) and BioGRID (https://downloads.thebiogrid.org/; Saccharomyces_cerevisiae_S288c-4.3.196). The accompanying notebook is available in Supplementary Data 1 as Yeast_Network_comparisons.ipynb. Gene annotation enrichment was performed using the 1D tool in Perseus (v.1.6.7.0). Annotation terms were filtered for 5% FDR (Benjamini–Hochberg correction) and a score above 0.

### Structure predictions

All structures were calculated on a Linux cluster utilizing up to 4 Nvidia A100 GPUs, 512 GB RAM and 72 CPUs. AlphaFold-Multimer version 2.2.0 (ref. ^47^) was used and predictions are based on full length sequences. An exception to this is the structure in Fig. 5a (iii) which was predicted using v2.1.1 and Fig. 5c,d, middle, which was predicted without the N-terminally processed targeting sequences. Global model confidence scores (ipTM+pTM) are weighted in favour of the interface score ipTM as described^47^. For the calculations of clusters in Supplementary Fig. 3 containing novel interactions and uncharacterized proteins, we filtered based on their total residue size below 2,500—an approximate limit that is set by the unified GPU memory size. For runs that did not succeed within 24 h or resulted in model confidences below 0.7 subclusters were selected and resubmitted. The final structures were included if they had a score above 0.7 (median scores above 0.85). Structures with not all submitted subunits clearly participating in a single complex interaction were excluded and Supplementary Fig. 9r,t were rerun with optimized stoichiometry. Molecular graphics were generated with UCSF ChimeraX (v1.4), developed by the Resource for Biocomputing, Visualization, and Informatics at the University of California, San Francisco, with support from National Institutes of Health R01-GM129325 and the Office of Cyber Infrastructure and Computational Biology, National Institute of Allergy and Infectious Diseases^66^. Alignments were generated using Jalview (v2.11.2.0)^67^ and the protein domain map with DOG 2.0 (ref. ^68^).

### Spotting assay

We generated the knockout strains by replacing the endogenous loci of CKA1 and CKA2 with the URA3 marker gene in the diploid wild-type strain BY4743 via standard LiAc transformation and PCR validation^69^. Haploid strains were retrieved by sporulation and tetrad dissection of diploid strains and 2:2 segregation was validated by replica plating on corresponding marker and mating type plates. We generated the galactose-induced overexpression plasmid pGAL1-YLR407W via Gibson cloning. Insert and plasmid backbone were generated by high-fidelity PCR amplification of the endogenous locus of YLR407W and the plasmid p415-GAL1, respectively. Plasmids were validated by sequencing and transformed into competent haploid wild-type strain BY4742 and corresponding haploid knockout strains. Spotting was carried out in 1:6 serial dilution series.

### Reporting summary

Further information on research design is available in the Nature Portfolio Reporting Summary linked to this article.

## Online content

Any methods, additional references, Nature Portfolio reporting summaries, source data, extended data, supplementary information, acknowledgements, peer review information; details of author contributions and competing interests; and statements of data and code availability are available at 10.1038/s41586-023-06739-5.

### Supplementary information

Supplementary Information This file contains Supplementary Figs. 1–4.

Reporting Summary

Supplementary Data 1 Analysis_Scripts

Supplementary Data 2 Interactome_Data

## Figures and Tables

**Extended Data Table 1:**
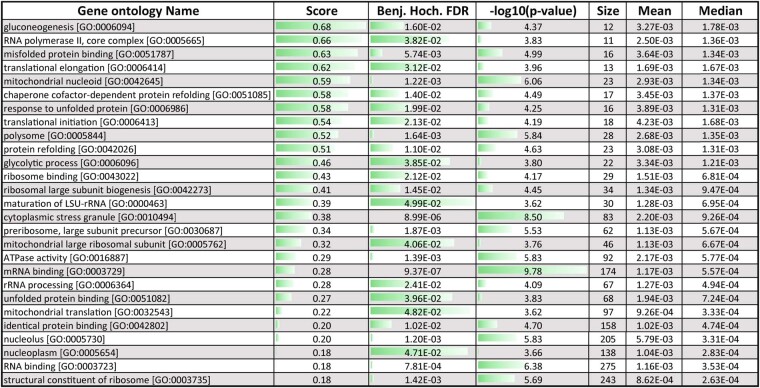
Gene ontology term enrichment

Gene ontology term enrichment on betweenness-centrality of nodes (proteins) in the network (1-dimensional annotation enrichment, two-sided, FDR < 5%, score > 0).

## Data Availability

All main mass spectrometry raw data and MaxQuant output tables have been deposited to the ProteomeXchange Consortium^70^ via the PRIDE partner repository with the dataset identifier PXD031940. The dataset is publicly available under www.yeast-interactome.org.

## Code availability

Custom code is available in Supplementary Data 1 as indicated in the relevant sections in Methods.
