# Supplementary material for: The social and structural architecture of the yeast protein interactome
Source: Nature. 2023 Nov 15;624(7990):192–200. doi: 10.1038/s41586-023-06739-5 (PMC10700138; doi:10.1038/s41586-023-06739-5)
Supplement: Supplementary file 1 — This file contains Supplementary Figs. 1–4. [file 41586_2023_6739_MOESM1_ESM.pdf]

nature portfolio

https://doi.org/10.1038/s41586-023-06739-5

Supplementary information

The social and structural architecture of the yeast protein interactome

In the format provided by the authors and unedited

Supplementary Figures

Michaelis et al.

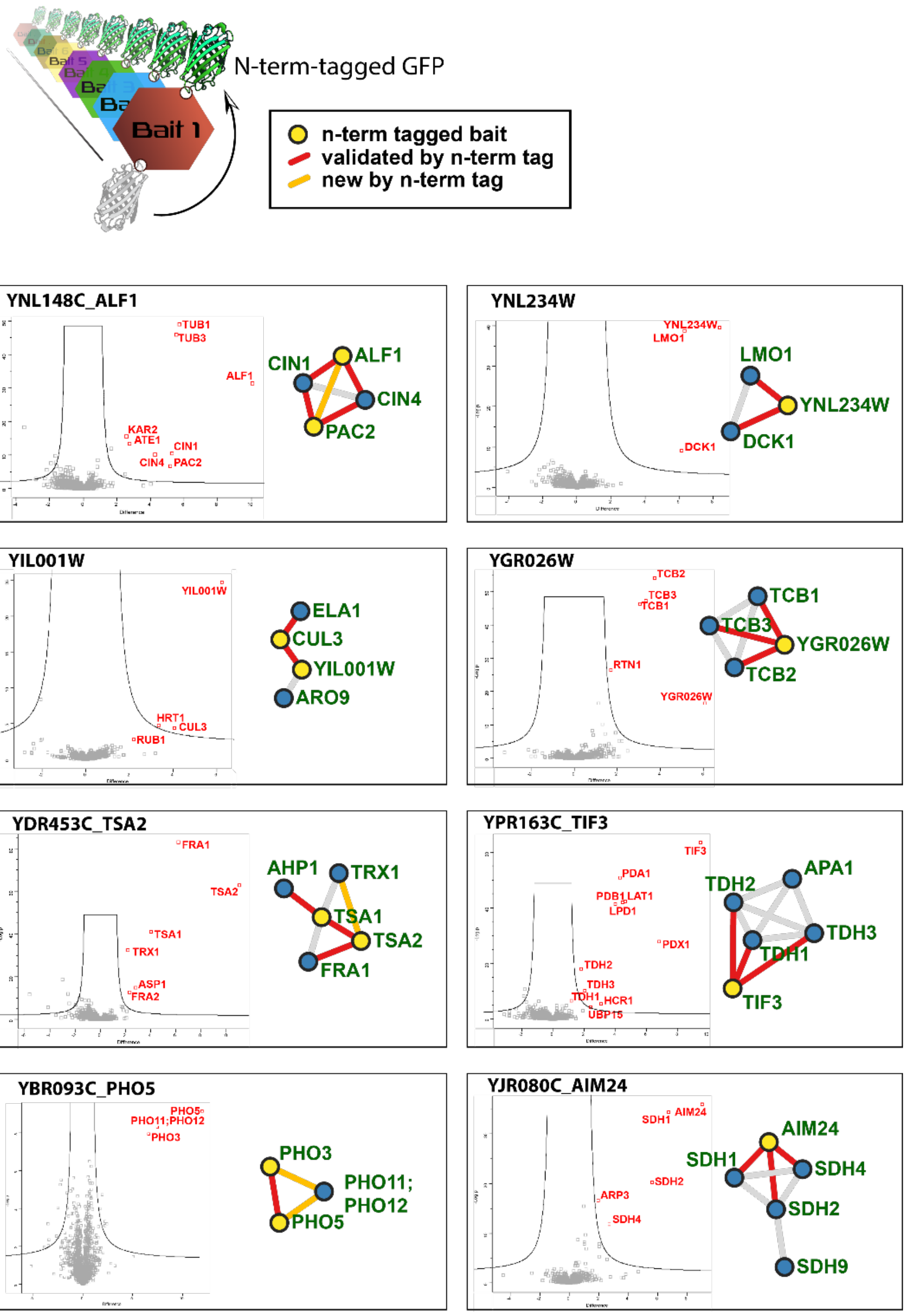

Supplementary Information Figure 1 (part 1/2) | Validation of selected clusters containing novel interactions or uncharacterized proteins using n-terminal GFP tagged baits. x-axis: log2(foldchange), y-axis: -log10(p-value); FDR=0.01.

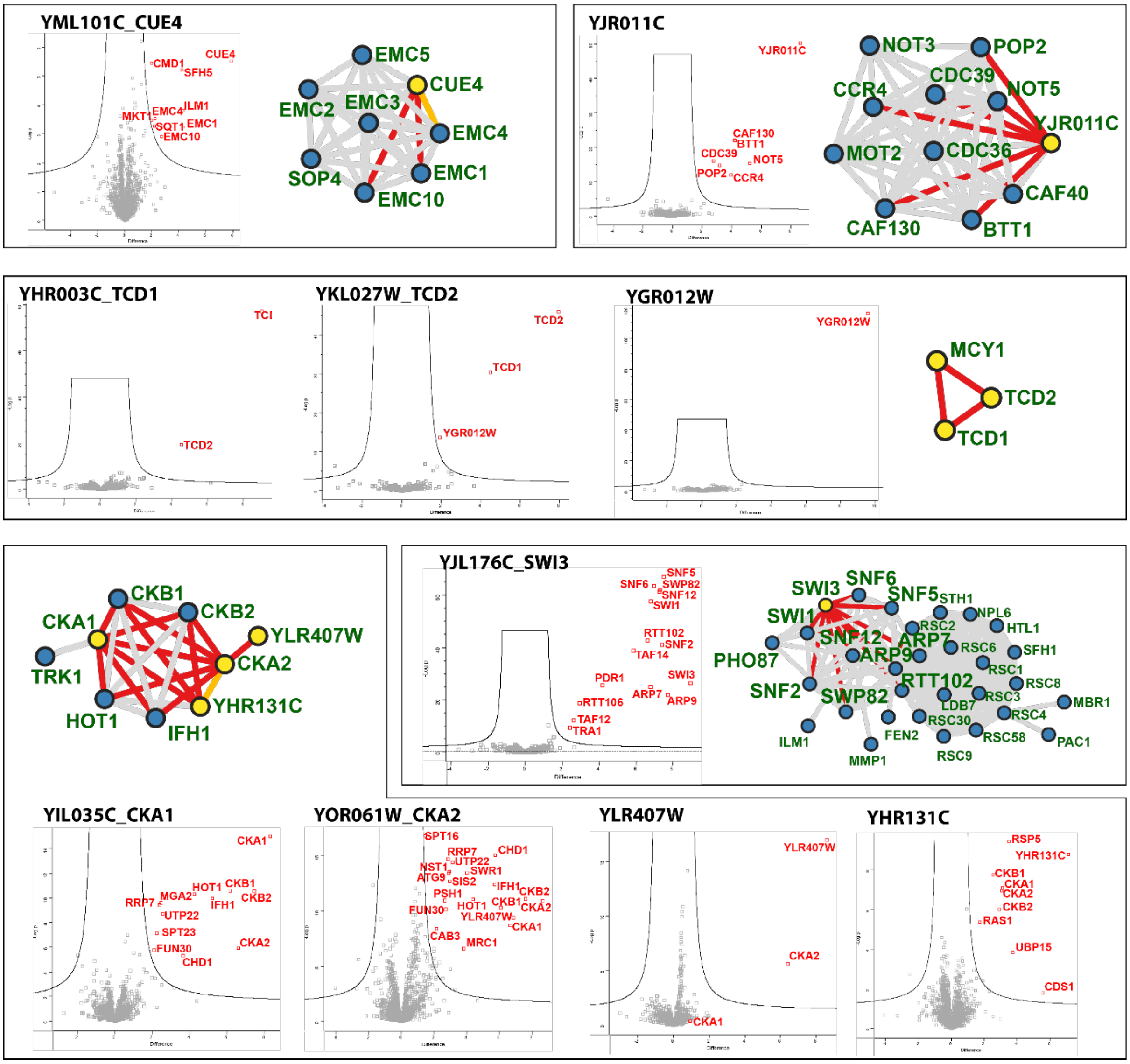

Supplementary Information Figure 1 (part 2/2) | Continuation of supplementary figure 1.

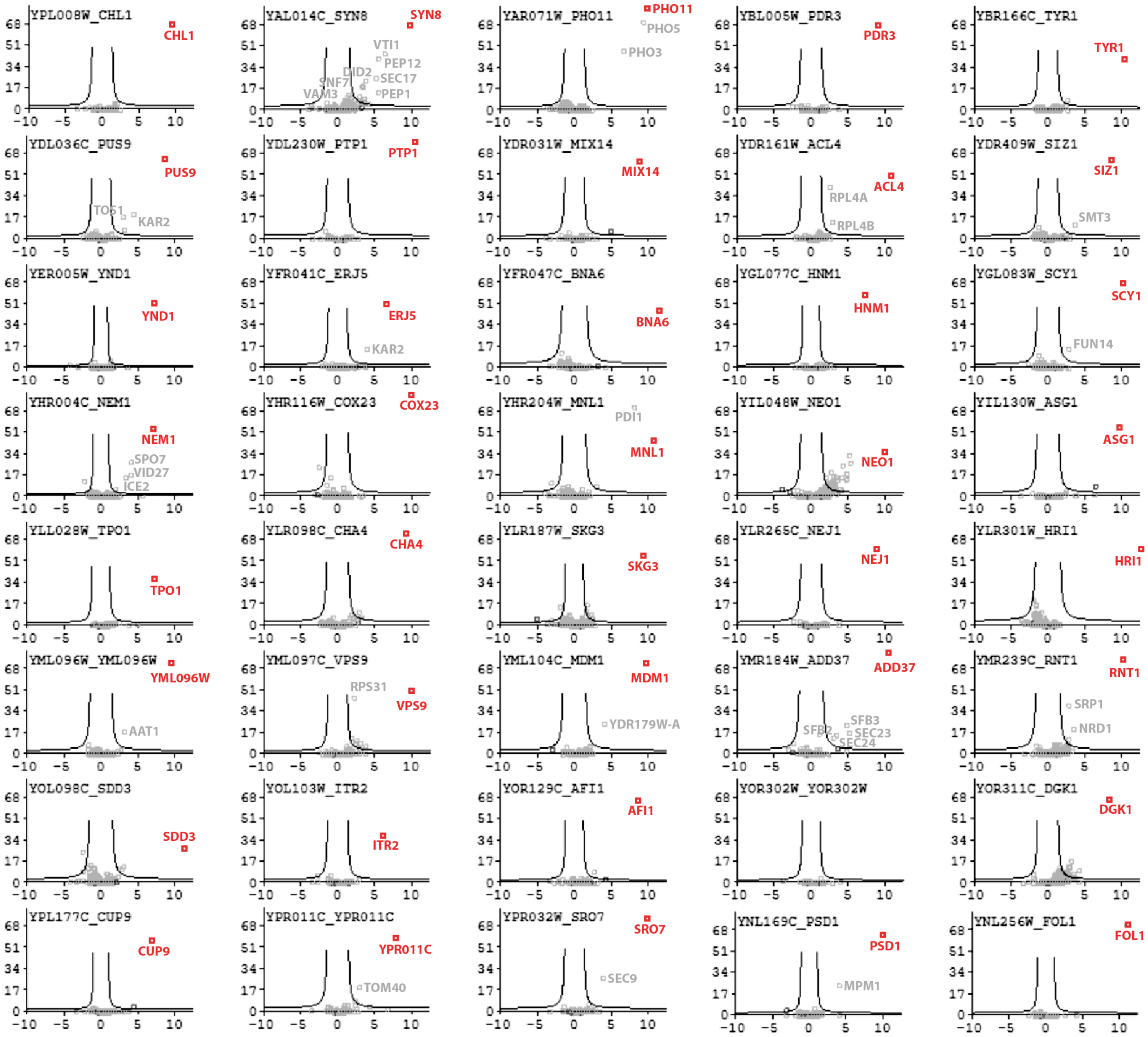

Supplementary Information Figure 2 (part 1/2) | Reciprocal pull-downs (N-terminal Nop1pr-GFP-tagged, Weill et al., 2018) of proteins without a significant interactor in C-terminally tagged main dataset (selection from Suppl. Fig. 5.). About half of the pull-downs identified significant interactors. Bait proteins samples proteins highlighted in red. x-axis: log2(foldchange), y-axis: -log10(p-value); FDR=0.01.

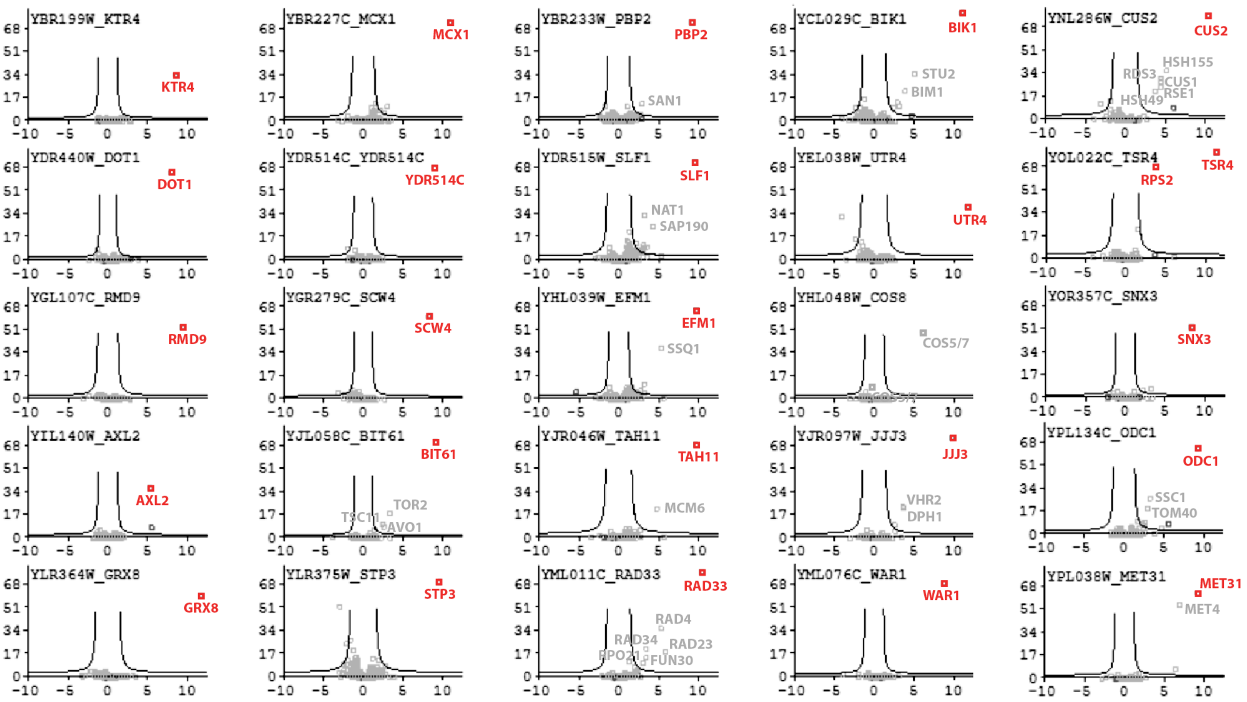

Supplementary Information Figure 2 (part 2/2) | Continuation of supplementary figure 2.

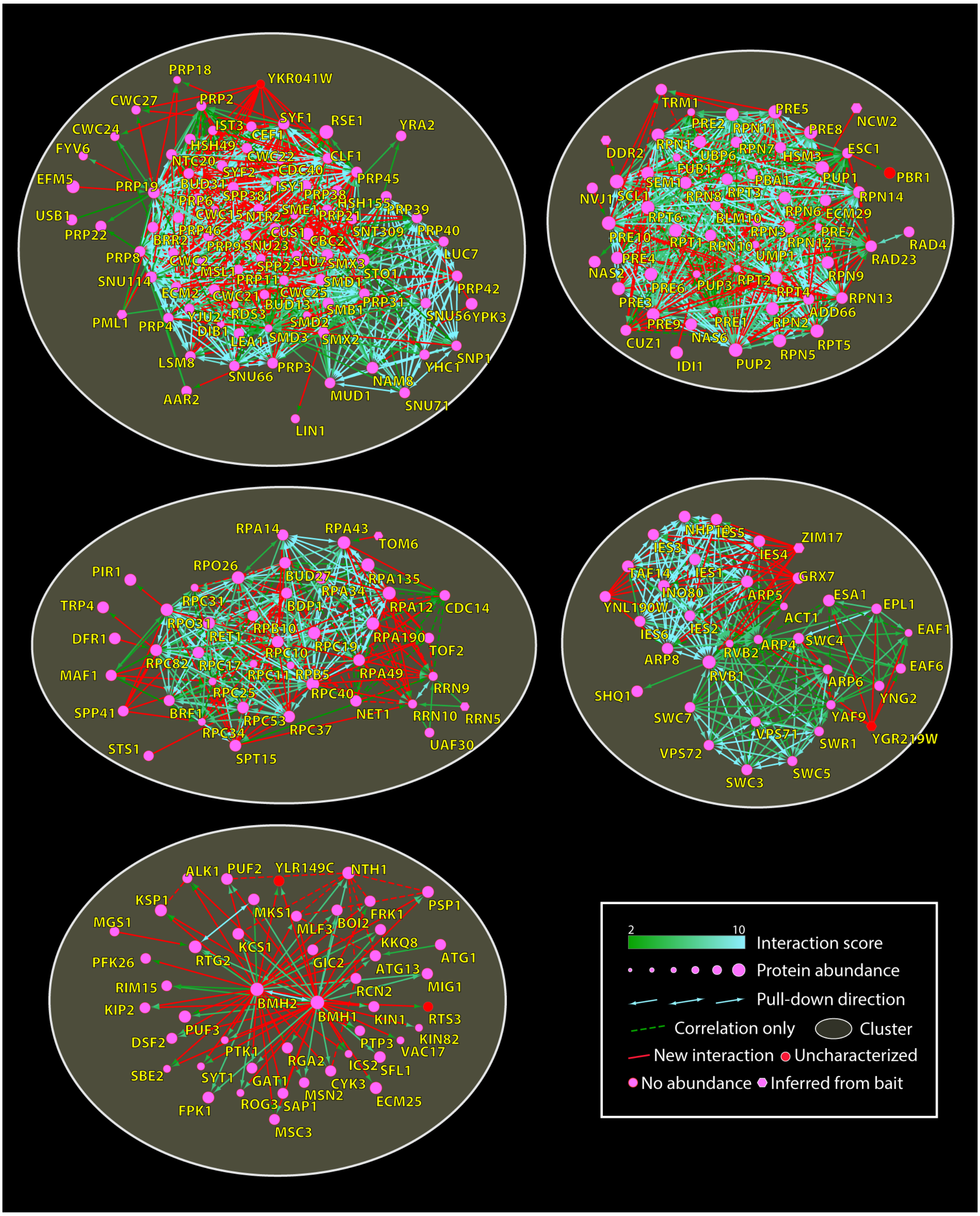

Supplementary Information Figure 3 (part 1/6) | Extended selection of clusters involving proteins with novel interactions and/or uncharacterized proteins supported by multiple layers of evidence.

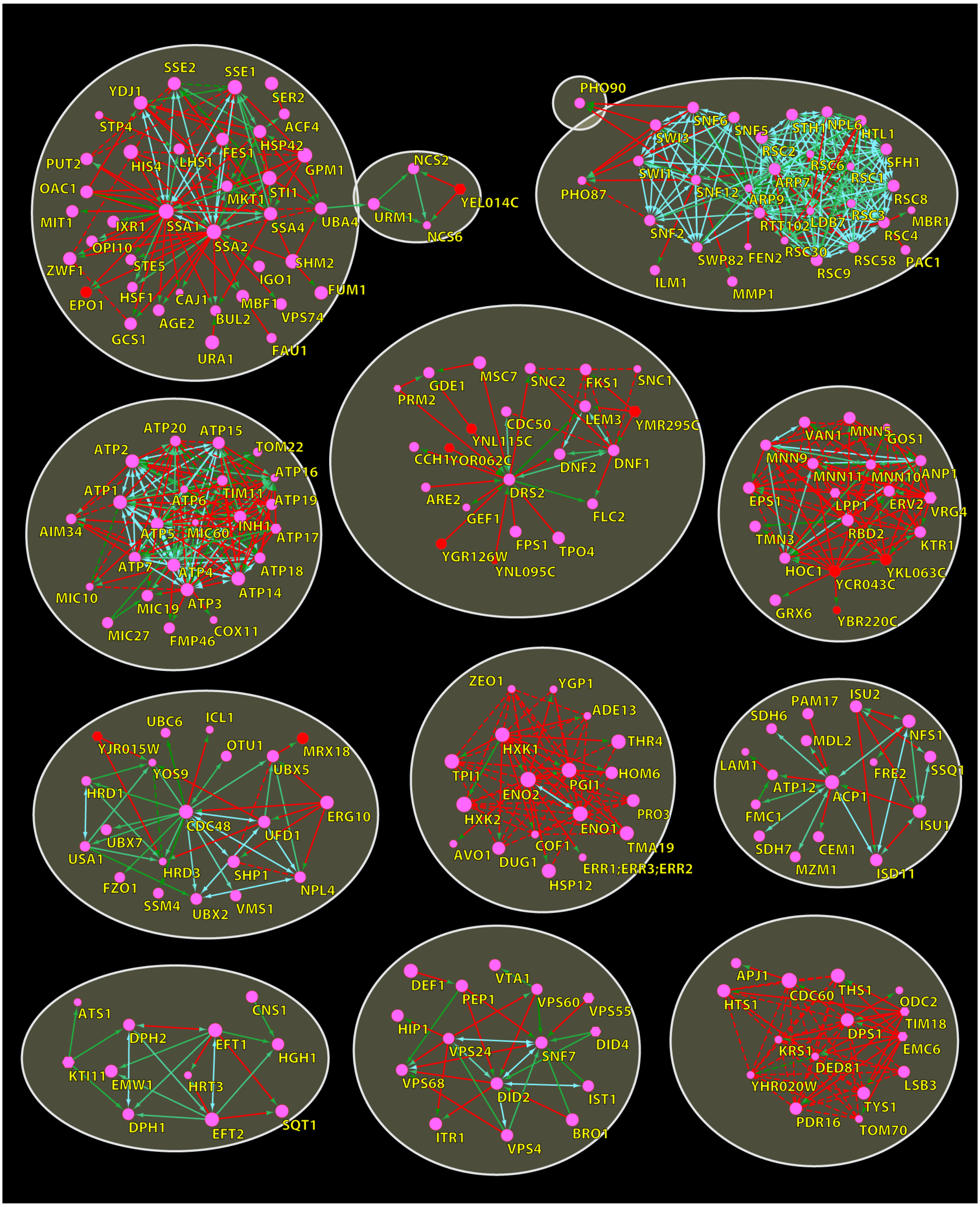

Supplementary Information Figure 3 (part 2/6) | Continuation of supplementary figure 3.

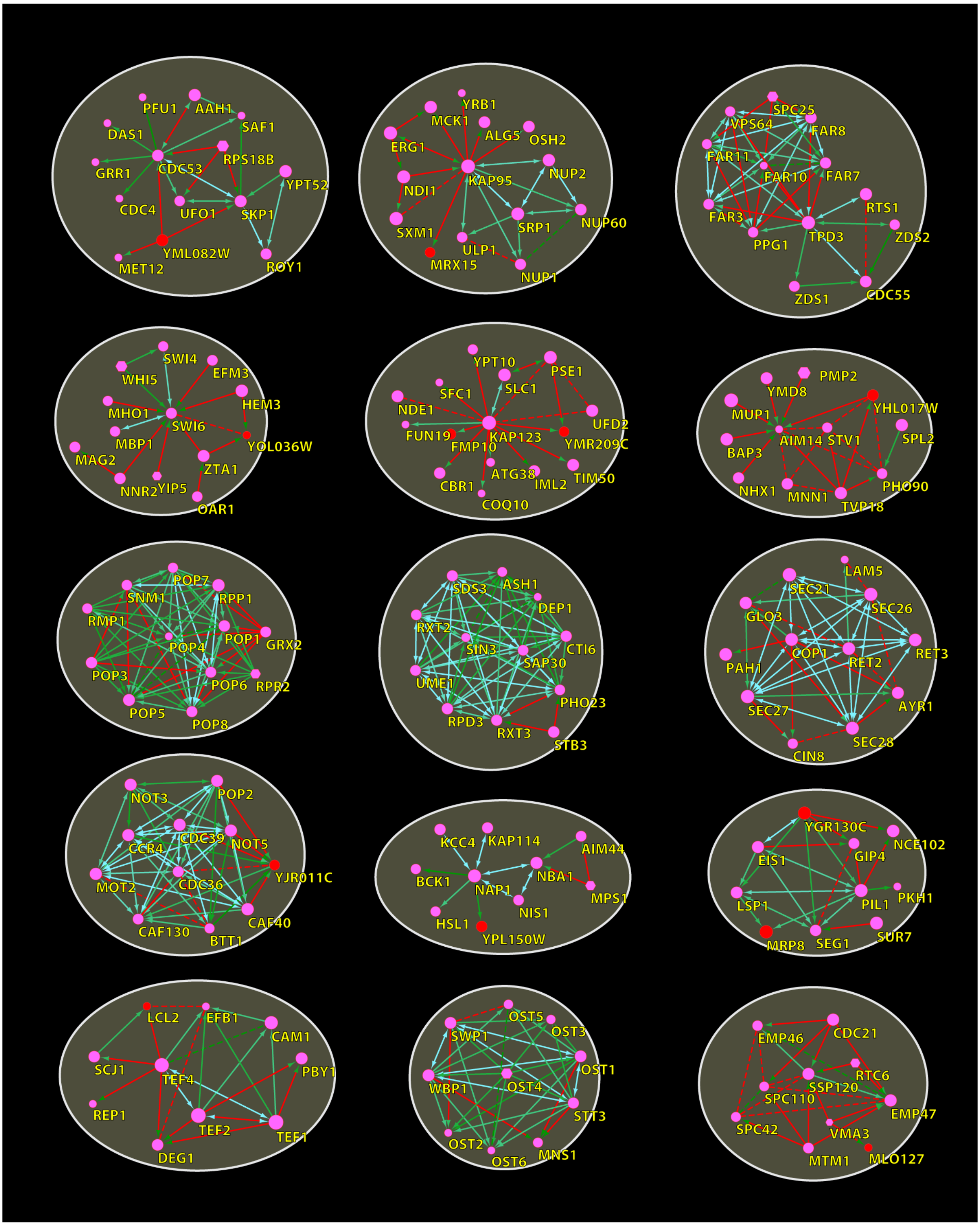

Supplementary Information Figure 3 (part 3/6) | Continuation of supplementary figure 3.

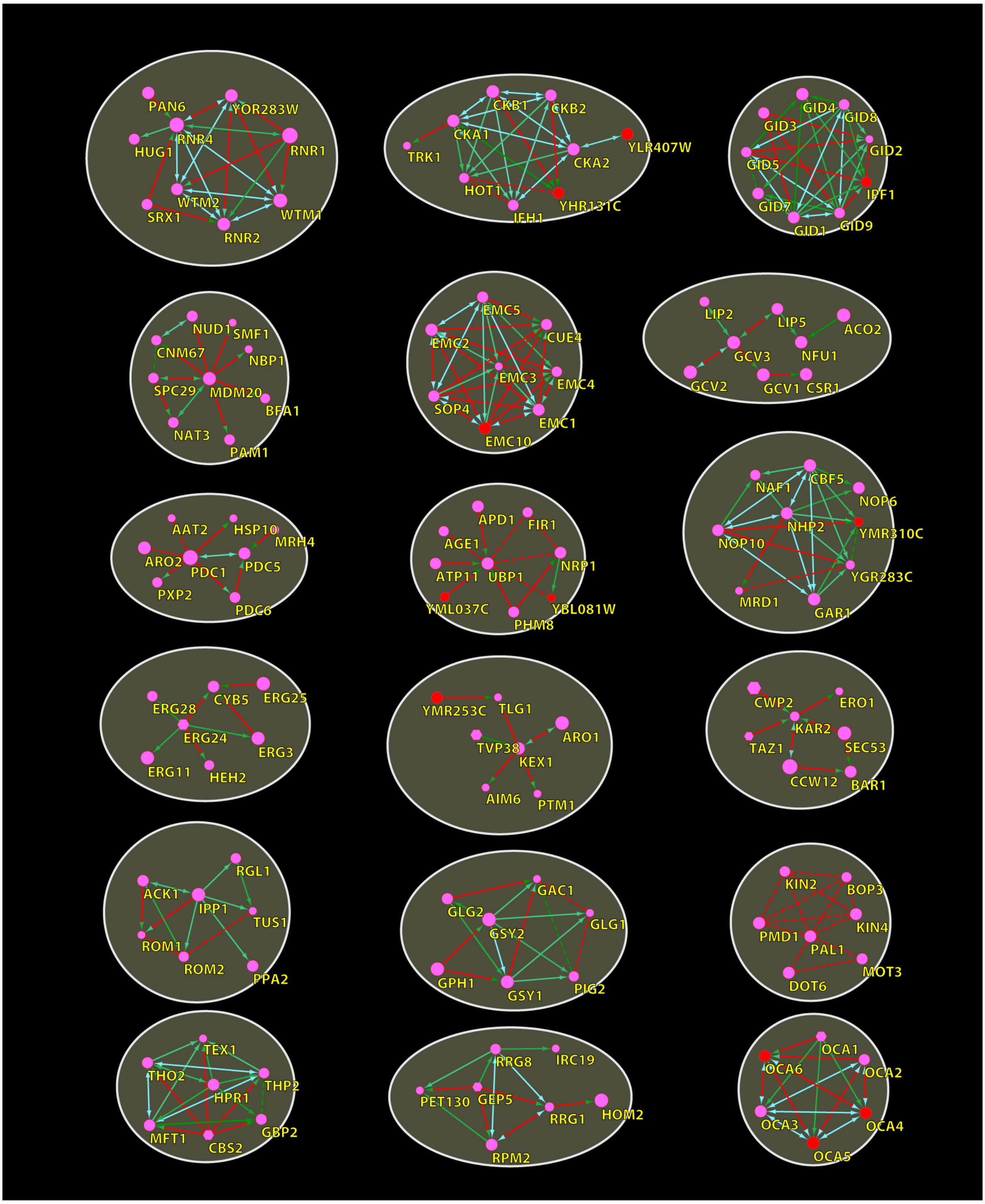

Supplementary Information Figure 3 (part 4/6) | Continuation of supplementary figure 3.

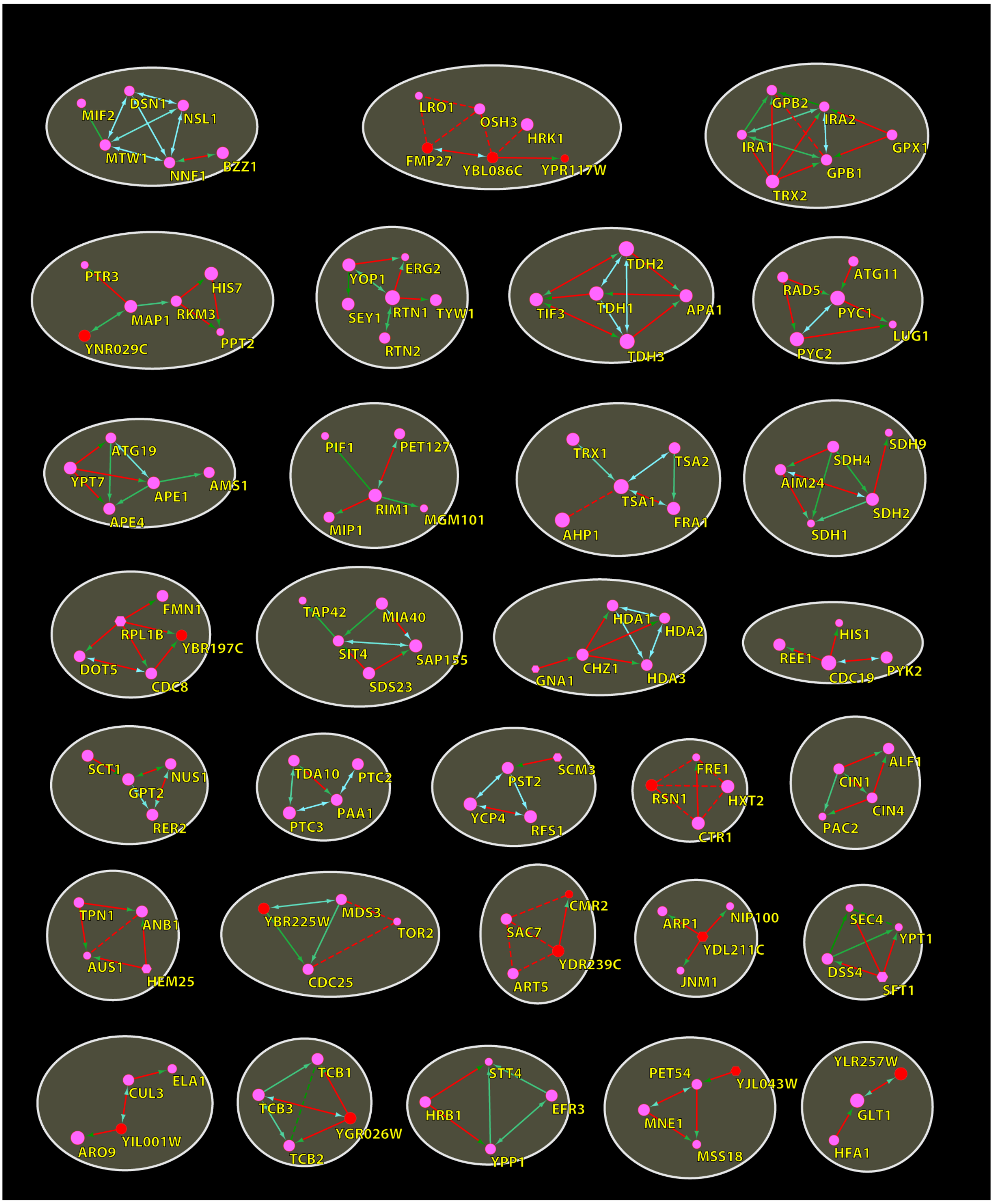

Supplementary Information Figure 3 (part 5/6) | Continuation of supplementary figure 3.

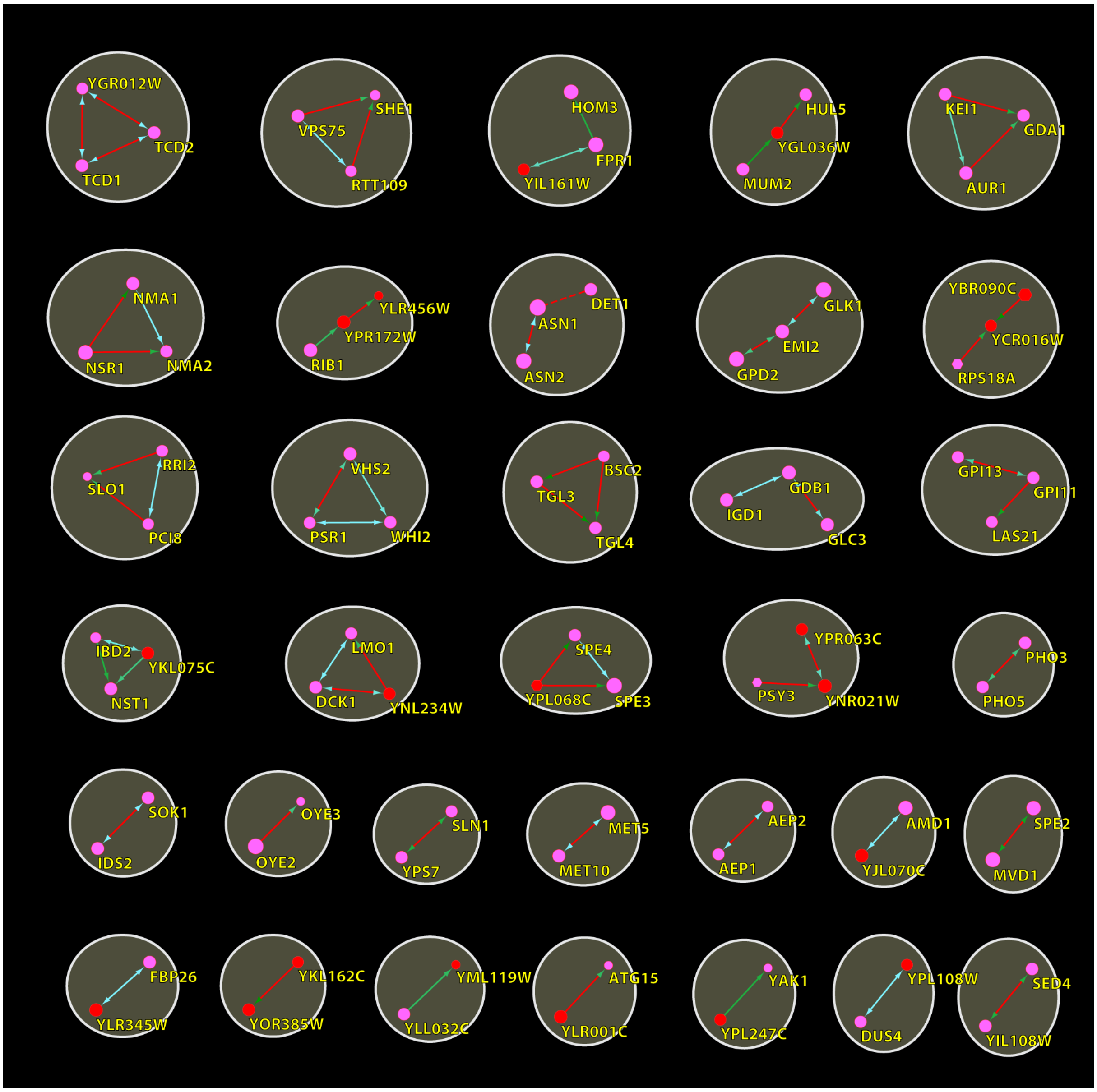

Supplementary Information Figure 3 (part 6/6) | Continuation of supplementary figure 3.

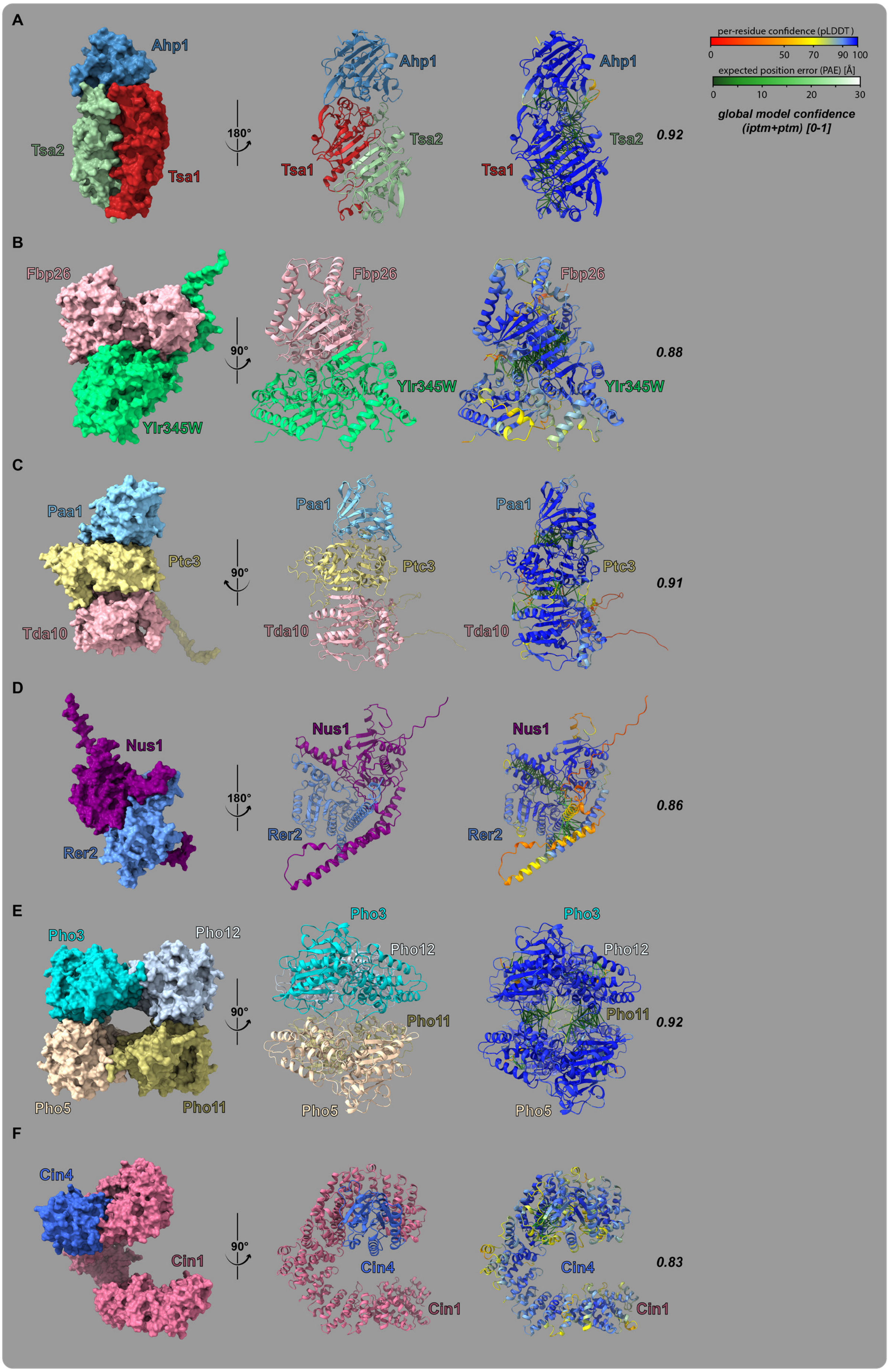

Supplementary Information Figure 4 (part 1/4) | Clusters and subclusters involving proteins with novel interactions and/or uncharacterized proteins.

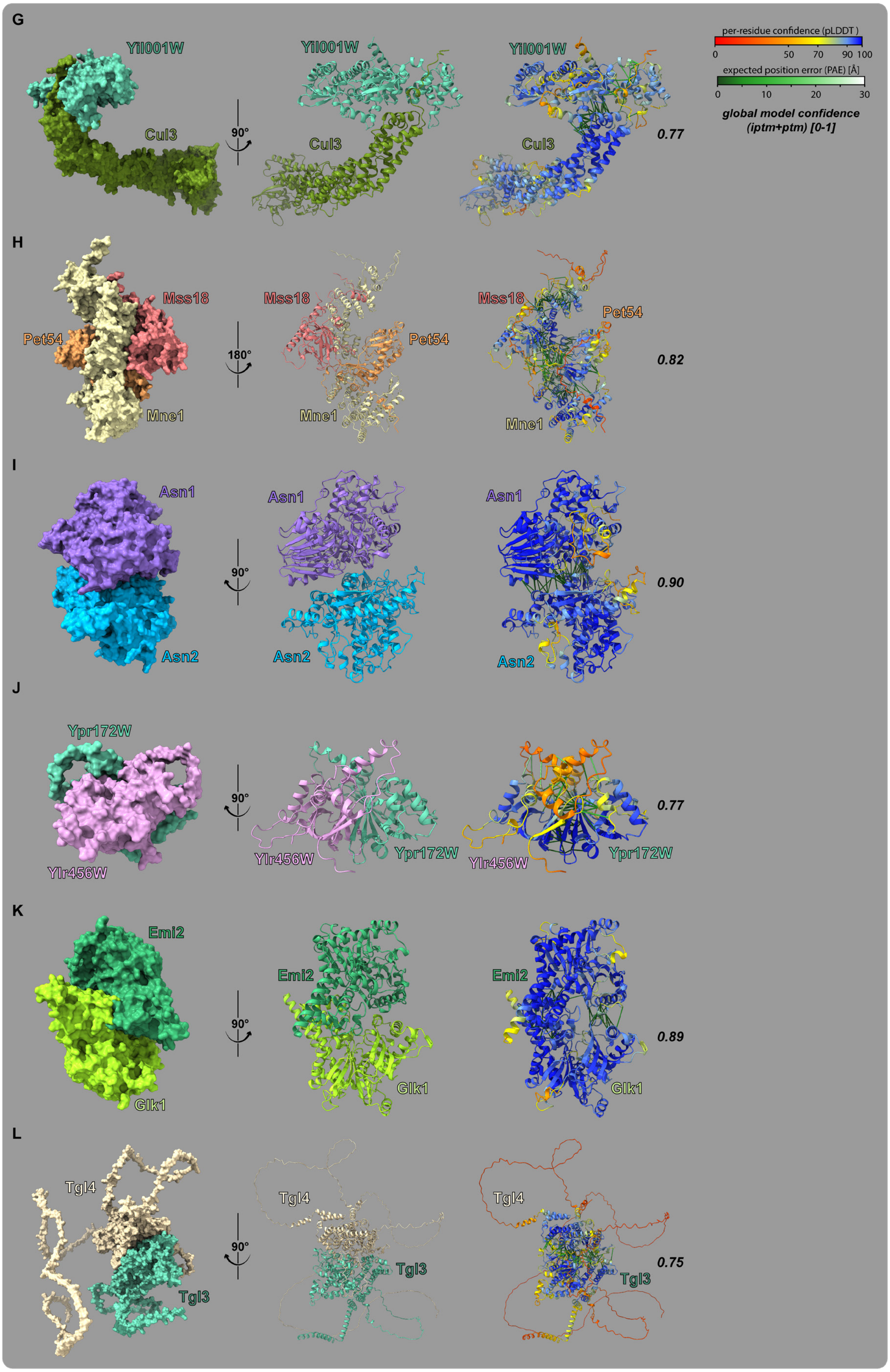

Supplementary Information Figure 4 (part 2/4) | Continuation of supplementary figure 4.

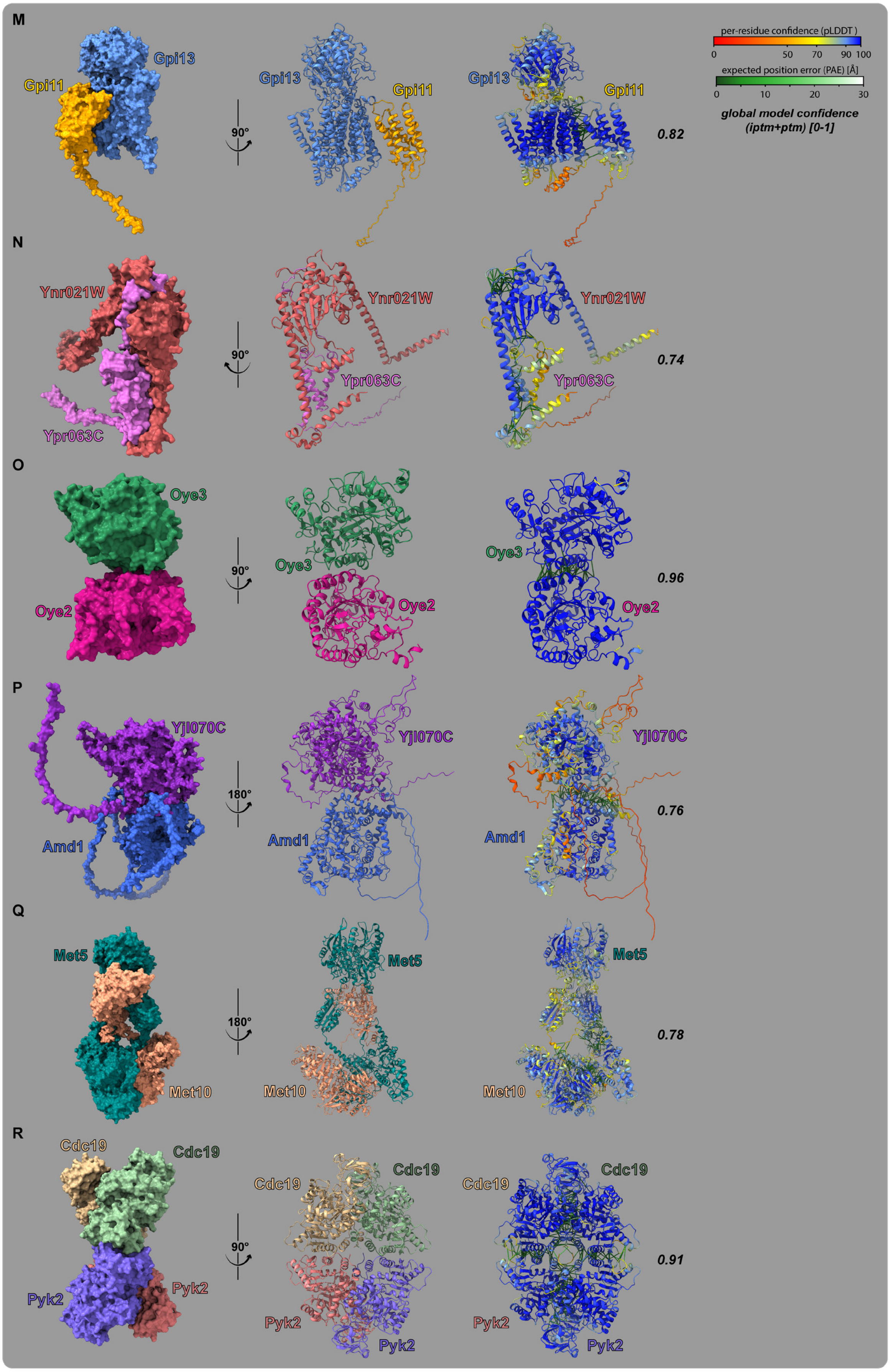

Supplementary Information Figure 4 (part 3/4) | Continuation of supplementary figure 4.

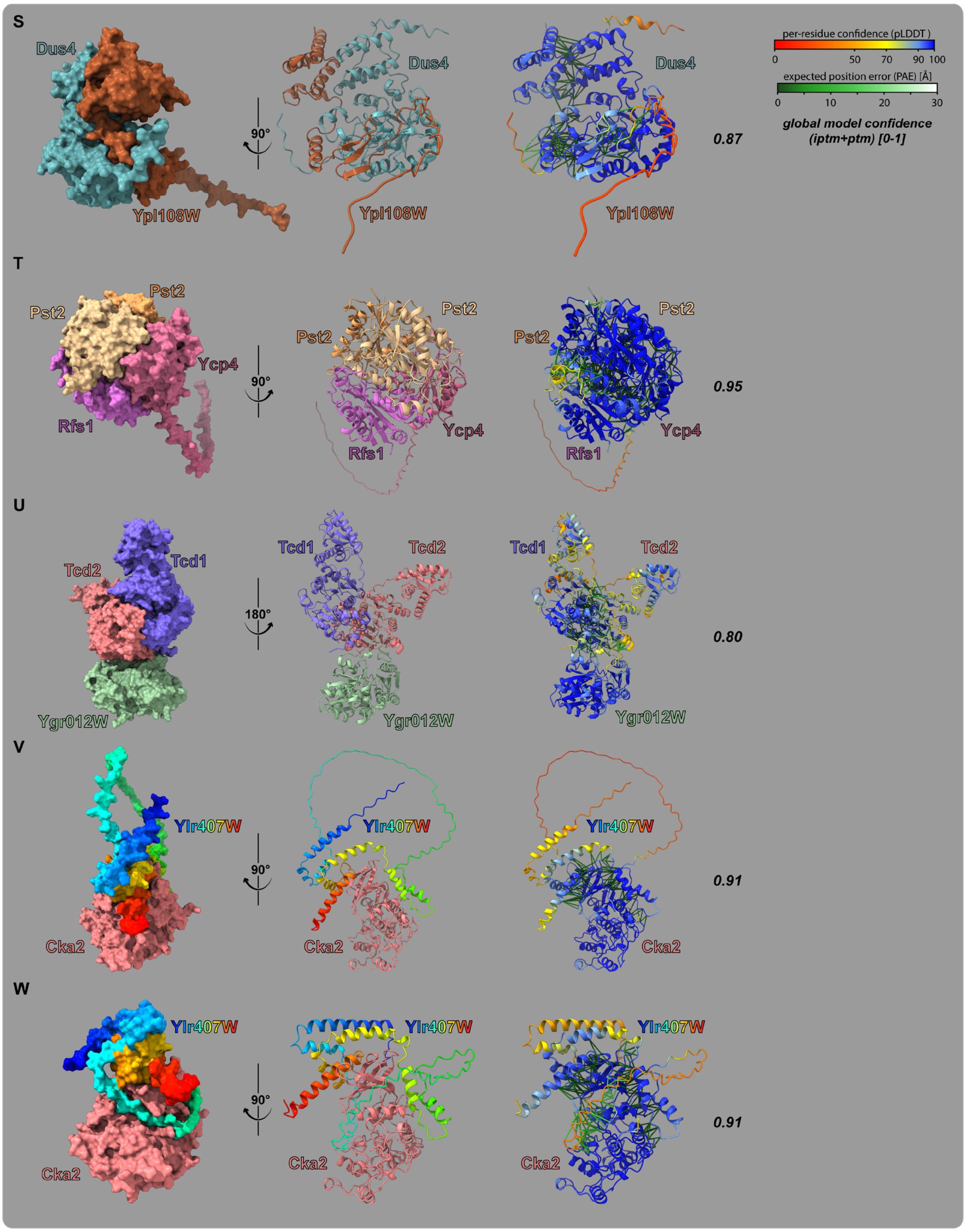

Supplementary Information Figure 4 (part 4/4) | Continuation of supplementary figure 4.
